# Quantitative hopanoid analysis enables robust pattern detection and comparison between laboratories

**DOI:** 10.1111/gbi.12132

**Published:** 2015-04-11

**Authors:** C-H Wu, L Kong, M Bialecka-Fornal, S Park, A L Thompson, G Kulkarni, S J Conway, D K Newman

**Affiliations:** 1 Division of Biology and Biological Engineering, California Institute of TechnologyPasadena, CA, USA; 2 Howard Hughes Medical InstitutePasadena, CA, USA; 3 Chemistry Research Laboratory, Department of Chemistry, University of OxfordOxford, UK; 4 Division of Geological and Planetary Sciences, California Institute of TechnologyPasadena, CA, USA

## Abstract

Hopanoids are steroid-like lipids from the isoprenoid family that are produced primarily by bacteria. Hopanes, molecular fossils of hopanoids, offer the potential to provide insight into environmental transitions on the early Earth, if their sources and biological functions can be constrained. Semiquantitative methods for mass spectrometric analysis of hopanoids from cultures and environmental samples have been developed in the last two decades. However, the structural diversity of hopanoids, and possible variability in their ionization efficiencies on different instruments, have thus far precluded robust quantification and hindered comparison of results between laboratories. These ionization inconsistencies give rise to the need to calibrate individual instruments with purified hopanoids to reliably quantify hopanoids. Here, we present new approaches to obtain both purified and synthetic quantification standards. We optimized 2-methylhopanoid production in *Rhodopseudomonas palustris* TIE-1 and purified 2Me-diplopterol, 2Me-bacteriohopanetetrol (2Me-BHT), and their unmethylated species (diplopterol and BHT). We found that 2-methylation decreases the signal intensity of diplopterol between 2 and 34% depending on the instrument used to detect it, but decreases the BHT signal less than 5%. In addition, 2Me-diplopterol produces 10× higher ion counts than equivalent quantities of 2Me-BHT. Similar deviations were also observed using a flame ionization detector for signal quantification in GC. In LC-MS, however, 2Me-BHT produces 11× higher ion counts than 2Me-diplopterol but only 1.2× higher ion counts than the sterol standard pregnane acetate. To further improve quantification, we synthesized tetradeuterated (D_4_) diplopterol, a precursor for a variety of hopanoids. LC-MS analysis on a mixture of (D_4_)-diplopterol and phospholipids showed that under the influence of co-eluted phospholipids, the D4-diplopterol internal standard quantifies diplopterol more accurately than external diplopterol standards. These new quantitative approaches permit meaningful comparisons between studies, allowing more accurate hopanoid pattern detection in both laboratory and environmental samples.

## Introduction

Hopanoids are bacterial membrane lipids from the pentacyclic triterpenoid family that have been identified in terrestrial, geothermal, freshwater, and marine environments (Pearson *et al*., 2007; Saenz *et al*., 2011; Ricci *et al*., 2014). The carbon skeleton of hopanoids can be preserved after diagenesis, resulting in hopanes, which have been proposed as environmental and ecological biomarkers (Ourisson & Albrecht, 1992; Brocks & Pearson, 2005). Indeed, the earliest fossil record of hopanes can be dated back to at least 1.6 billion years (Gyr) ago (Brocks *et al*., 2005; Rasmussen *et al*., 2008), and spikes in the 2-methylhopane index correlate with ancient ocean anoxic events (Knoll *et al*., 2007).

To better interpret the sedimentary hopane record, a thorough understanding of the biological functions and sources of hopanoids, as well as the environmental factors that govern their production, is required. Traditionally, the relative abundance of environmental hopanoids was interpreted in the context of what was known about hopanoid production by select strains in the laboratory (Talbot *et al*., 2008). With the application of genomic and genetic analyses, environmental hopanoid measurements could be correlated with the presence of genes responsible for hopanoid biosynthesis. The capacity for hopanoid production by diverse groups, including methane-oxidizing bacteria, iron-reducing bacteria, cyanobacteria, alphaproteobacteria, and acidobacteria, is well documented (Fischer *et al*., 2005; Pearson *et al*., 2009; Welander *et al*., 2010; Ricci *et al*., 2014). However, until now, quantification of hopanoid production, transport, burial, and preservation has been relative both within and between laboratories (i.e., the abundance of compound X is two times the abundance of compound Y), due to the fluctuation of ionization between hopanoid variants and instrumentation, as well as different analytical methods. Such variance has hindered the assemblage of meaningful metadata sets to assess whether hopanoids are reliable indicators for particular environmental processes.

Among the different hopanoid variants, 2-methylhopanoids have drawn close scrutiny. Originally thought to be produced mainly by cyanobacteria and thereby correlated with the capacity for oxygenic photosynthesis (Summons *et al*., 1999), they were later found to be produced in high abundance by the anoxygenic phototroph, *Rhodopseudomonas palustris* TIE-1, and the plant symbiont, *Bradyrhizobium japonicum* (Rashby *et al*., 2007; Talbot *et al*., 2007). Molecular genetic studies, using *R. palustris* TIE-1 as a model hopanoid-producing organism, revealed that *hpnP* is the gene responsible for methylation at the C-2 position, and its expression is upregulated by the general stress response sigma factor EcfG (Welander *et al*., 2010; Kulkarni *et al*., 2013). Metagenomic and clone library sequence analyses demonstrated that modern *hpnP*-containing microbes tend to occupy sessile niches characterized by low oxygen, high osmolarity, and nitrogen fixation, such as those found in the rhizosphere and microbial mats (Ricci *et al*., 2014). To determine whether 2-methylhopanoids improve fitness under these (or any other) conditions, absolute and accurate hopanoid quantification methods are essential.

Analytical methods with improved sample separation and detection sensitivity, such as GC-MS or LC-MS, have been developed for hopanoid detection (Talbot *et al*., 2007; Sessions *et al*., 2013). Despite the ability to separate and detect different hopanoids from complex mixtures, quantification has been hindered due to the difference in ionization efficiencies exhibited by the hopanoids (Schulenberg-Schell *et al*., 1989; Gibson *et al*., 2014). Common sterol-like internal standards such as androsterone or pregnane acetate have been used to quantify hopanoids, but whether their detection efficiency is the same as that of the hopanoids they are meant to standardize was untested (Sessions *et al*., 2013). Furthermore, potential ion-suppression effects arising from the co-elution of hopanoids with other compounds could render non-hopanoid internal standards ineffective. Accordingly, we reasoned that the availability of purified hopanoids as external standards and synthetic deuterated hopanoids as internal standards would facilitate accurate quantification of hopanoids in biological and environmental samples.

While other groups have made such standards by purifying 2-methyl tetrahymanol, aminotriol, bacteriohopanetetrol (BHT), and its glucosamine derivatives, or synthesizing dideuterated BHT-glucosamine (Bisseret & Rohmer, 1989; Moreau *et al*., 1995; Bravo *et al*., 2001; Pan *et al*., 2005, 2007; Pan & Vincent, 2008), dideuterated BHT-glucosamine is limited in its technical application, and previous studies did not focus on 2-methylhopanoids. To fill these gaps, we engineered *R. palustris* TIE-1 to maximize production of 2-methylated hopanoids and developed synthetic approaches to obtain milligram quantities of tetradeuterated hopanoids as standards for quantification by mass spectrometry. Here, we show how use of these new standards (in contrast to conventional analytical methods) can significantly change the conclusions one would derive about the abundance of particular hopanoids in a sample. We close with a discussion of the importance of standardizing hopanoid analytical approaches for earth science applications.

## Methods

### Bacterial strains and chemicals

Two strains of *R. palustris* TIE-1 were used in this study, the wild-type (WT) strain and a mutant strain (DKN1283) with an IPTG-inducible *hpnP* gene integrated into the genome. Detailed protocols for making DKN1283 are described in the Supporting Information. A typical starting culture was prepared by inoculating a single colony of *R. palustris* TIE-1 grown on YPMS-agar plates (0.3% yeast extract, 0.3% peptone, 50 mm morpholinepropanesulfic acid (MOPS), 5 mm sodium succinate, pH 7.0, 1.2% (w/v) agar) into 10 mL of YPMS and grown at 30 °C, 250 rpm for ∼7 days. IPTG was from OmniPur. MOPS, sodium succinate, methionine, squalene, vitamin B12, pyridine (HPLC grade), acetic anhydride (ReagentPlus), androsterone, 5β-pregnane-3α,12α-diol-20-one diacetate (pregnane acetate), silica gel (70–230 mesh, 63–200 μm, pore size 60 Å, technical grade), and Amberlite IR-120 (hydrogen form) were purchased from Sigma-Aldrich. Celite 545 was purchased from EMD Millipore. Solvents for total lipid extraction and silica gel purification (hexanes (Hex), ethyl acetate (EtOAc), and dichloromethane (CH_2_Cl_2_) were environmental grade and for HPLC purification [methanol (MeOH), isopropanol (IPA), and water] were HPLC grade, all purchased from Alfa Aesar. Phospholipids (PC, PG, and PS) were purchased from Avanti Polar Lipids.

### Optimization of growth conditions for 2-methylhopanoid production

In Olympus flat bottom 96-well tissue culture plates (Genesee Scientific, San Diego, CA, USA), 2.5 μL of starting cultures of *R. palustris* TIE-1 WT and DKN1283 was inoculated into 250 μL per well of media containing YPMS with or without 5 mm methionine, 0.7 μm vitamin B12, or 10 mm acetate, and grown in an Intellus incubator (Percival Scientific, Perry, IA, USA) at 30 °C under light (with 60 W incandescent and 17 W fluorescent light bulbs). For growth without light, the plates were wrapped in aluminum foil. For DKN1283, IPTG (1 mm final concentration) was added ∼1 day after inoculation. For YPMS containing squalene (10 mm final concentration), a 1:1 squalene: THF was added ∼1 day after initial inoculation. The growth (OD_600_) of cultures was monitored in a Synergy4 plate reader (BioTek, Winooski, VT, USA). The outer wells of 96-well plates were not used but filled with 250 μL of YPMS. There were four technical replicates for each growth condition. To analyze the hopanoid content, cells from four wells of culture in the same conditions were combined, spun down, resuspended in 50 μL of water, and transferred into GC vials with 400-μL size glass inserts. MeOH (125 μL) and CH_2_Cl_2_ (62.5 μL) were added, and the vials were capped and sonicated in a B2500A-DTH sonicator (42 kHz, RF Power 85 W, VWR, Bridgeport, NJ, USA) for 30 min at RT. The total lipids were extracted by addition of ∼200 μL of CH_2_Cl_2_ and mixed by pipetting. The lower organic layer that contained the hopanoids was collected, dried in a 60 °C oven, and acetylated in 100 μL of 1:1 pyridine: acetic anhydride (Ac_2_O) for 30 min at 60 °C. The acetylated hopanoids were analyzed with a Restek Rxi-XLB column (30 m × 0.25 mm × 0.10 μm) in a Thermo Scientific TraceGC coupled with ISQ mass spectrometer using previously described protocols (Welander *et al*., 2009).

To optimize the growth conditions to achieve maximum 2-methylhopanoid production at a larger scale, 2 L of medium containing 1 mm IPTG, 0.3% yeast extract, 0.3% peptone, and different concentrations of MOPS (0, 10, 20, 50 mm), succinate (5, 10, 20 mm), acetate (10, 20, 40 mm), or methionine (0.1, 1 mm) was prepared. The medium without IPTG or methionine was adjusted to pH 7.0 with 10 m NaOH and sterilized by autoclaving. Stock solutions of IPTG (1 m) and methionine (250 mm) were 0.2 μm filter sterilized and added along with the bacterial inoculum. Autoclaved water was used to adjust the final volume to 2 L.

**Figure 2:**
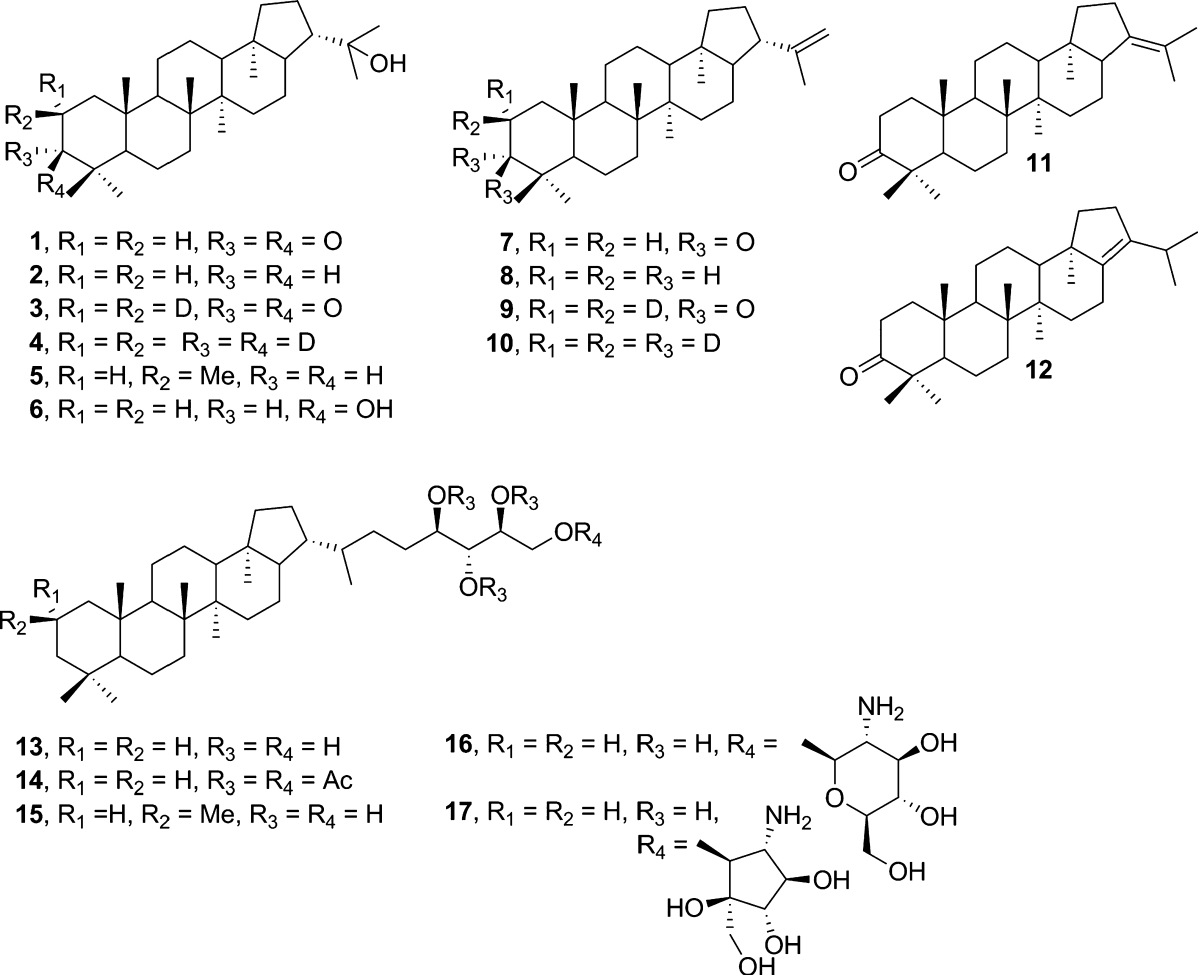
Structures of natural and synthetic hopanoids mentioned in the text. 1: hydroxyhopanone, 2: diplopterol (Dip), 3: 2,2-D_2_-hydroxyhopanone, 4: 2,2,3,3-D_4_-diplopterol, 5: (2*S*)-2-Methyl diplopterol (2Me-Dip), 6: (3*S*)-3-Hydroxydiplopterol, 7: 3-Oxo-diploptene, 8: diploptene, 9: 2,2-D_2_-3-Oxo-diploptene, 10: 2,2,3,3-D_4_-diploptene, 11: 3-Oxo-21-ene-deoxy-hopane, 12: 3-Oxo-17-ene-deoxy-hopane, 13: bacteriohopanetetrol (BHT), 14: 32,33,34,35-*tetra-O-*acetyl bacteriohopanetetrol (BHT-4Ac), 15: 2-Methyl bacteriohopanetetrol (2Me-BHT), 16: BHT-glucosamine, 17: BHT-cyclitol ether.

One milliliter of bacterial culture was inoculated into 600 mL of YPMS, 30 °C, 250 rpm for 3 days before inoculation into 2 L media (1:100 dilution). The 2 L culture was shaken at 180 rpm, 30 °C with light for phototrophic growth in an Innova44 New Brunswick Biological Shaker. This culture turned pink in 3 days and reached late stationary phase in 7 days with OD_600_ ∼0.9–1.4 (DU 800 Spectrophotometer, Beckman Coulter, Indianapolis, IN, USA). Cells were harvested by centrifugation at 7000 *g* for 7 min at 4 °C. The wet cell paste (dark burgundy) was stored at −20 °C before total lipid extraction and GC-MS analysis. The growth condition for maximal 2Me-BHT (**15,** Fig.2) production was selected and scaled up to 48 L cultures (in 24 2-L flasks) for each biological replicate.

### Large-scale total lipid extraction

Total lipid extract (TLE) from *R. palustris* TIE-1 strain DKN1283 was prepared using the Bligh–Dyer method (Bligh & Dyer, 1959) and modified for large-scale extraction. In a 4-L glass beaker, cell paste was suspended in water to reach a final volume of 5 mL suspension per gram of wet cell paste. Then, the equivalent of 12.5 mL methanol (MeOH) and 6.25 mL CH_2_Cl_2_ per gram of wet cell paste were added in sequence and stirred for 1 h before sonication for a total pulse time of 1 h while stirring (1/8 inch tip, power output 3.5, 1 s on, 4 s off, 4 °C, Sonic Dismembrator 550; Fisher Scientific, Pittsburgh, PA, USA). After the sonicated sample was warmed to room temperature (RT), CH_2_Cl_2_ was added at a volume ratio of sample: CH_2_Cl_2_ = 4:3. After gentle stirring, the sample was allowed to settle for a few hours to overnight, permitting the organic and aqueous layer to separate. The bottom organic layer (dark brown) was collected and dried using a rotary evaporator. A typical dark green TLE was obtained.

### Hopanoid purification by silica gel and HPLC

For silica gel purification, TLE was dissolved in CH_2_Cl_2_ and adsorbed on to Celite 545 (4 g per 1 g of TLE) using a rotary evaporator. About 7 g of Celite-adsorbed TLE was loaded onto a column packed with ∼300 mL of silica gel (∼25 × 4 cm) pre-equilibrated with hexane (Hex). The column was eluted in sequence of ∼450 mL Hex, ∼600 mL 3:1 hexanes:EtOAc, and ∼200 mL pyridine. The presence of diploptene, diplopterols, and BHTs in each respective elution was monitored by TLC plates and visualized by molybdate staining (10 g ammonium molybdate, 360 mL water, 40 mL 9 m H_2_SO_4_) after heating on a hot plate (diploptene *R*_f_ ∼ 0.97, (2Me)-diplopterol (**2** and **5**) *R*_f_ ∼ 0.51 in 5:1 hexanes:EtOAc, and (2Me)-BHT *R*_f_ ∼ 0.88 in 1:8:10 water:IPA:EtOAc). As the solubility of (2Me)-BHT (**13** and **15**) is too low for downstream HPLC purification, the sample was acetylated to increase solubility. The fractions containing (2Me)-BHT were dried using a rotary evaporator and acetylated in 20 mL pyridine and 20 mL acetic anhydride at 60 °C for ∼1.5 h. The acetylated BHTs were dried using the presence of Celite and packed onto a silica gel column as previously described. The column was equilibrated with ∼500 mL of 8:1 hexanes:EtOAc and eluted with ∼500 mL of 5:1 hexanes:EtOAc and then ∼500 mL of 4:1 hexanes:EtOAc. The presence of (2Me)-BHT-4Ac was monitored by the same TLC method as above (*R*_f_ ∼ 0.21 in 5:1 hexanes:EtOAc).

Further separation between methylated and unmethylated hopanoids was carried out using reverse-phase HPLC (Phenomenex Luna C18(2), 100 Å, 5 μm, 250 × 21.2 mm column coupled with a Shimadzu LC-8A preparatory pump and a Beckman SC 100 fraction collector). The silica gel-purified 2Me-diplopterol samples were dried and redissolved at 1 g mL^−1^ of CH_2_Cl_2_ and about 100 mg was injected into the HPLC, pre-equilibrated in 95:5 MeOH:water, and eluted with the same solvent at 20 mL min^−1^. The silica gel-purified 2Me-BHT-4Ac samples were purified by HPLC as above except the column was pre-equilibrated in 65:35 IPA:water and eluted with the same solvent at 12 mL min^−1^.

HPLC-purified BHT-4Ac (**14**) and 2Me-BHT-4Ac samples were collected and dried using a rotary evaporator. The samples were de-acetylated by dissolution in 1:1 CH_2_Cl_2_:MeOH with a substoichiometric amount of sodium methoxide and incubated with stirring at RT overnight. The products were passed through an Amberlite IR-120 column (hydrogen form, washed extensively with water, then dried with acetone, and packed in MeOH) and eluted with MeOH to exchange the by-product sodium acetate to acetic acid, which was subsequently removed along with solvent under vacuum using a rotary evaporator.

A high-precision balance (Sartorius, *d* = 0.001 mg) was used for weighing samples <10 mg, otherwise a Mettler Toldedo balance (*d* = 0.1 mg) was used. Characterization (e.g., ^1^H and ^13^C NMR, melting point, optical rotation/chirality, infrared, high-resolution mass spectrometry) of the purified hopanoids is included in the Supporting information. LC-MS analyses for purified hopanoids and sterol standards were carried out according to published protocols and included in SI (Malott *et al*., 2014).

### Synthesis and characterizations of hopanoids

Detailed experimental procedures used in the synthesis of the different C_30_ hopanoids, and their characterization, are included in the Supporting Information. Briefly, hydroxyhopanone was extracted from Dammar resin according to a published procedure (Dunstan *et al*., 1957). Diplopterol (**2**, Fig.2) and diploptene (**8**) can also be obtained from hydroxyhopanone (**1**). For diplopterol, diethylene glycol (50 mL) and hydrazine hydrate (3.6 mL) were added to hydroxyhopanone (500 mg, 1.13 mmol) in a glass flask and heated under reflux for 2 h. The excess of hydrazine and water were removed by distillation. Potassium hydroxide (1.3 g) was added, and the solution was heated to 200–210 °C for 6 h or overnight. After dilution with water, the product was extracted using an ether/water extraction. Evaporation of diethyl ether afforded a solid, which was crystallized from acetone–methanol to afford diplopterol as colorless solid (462 mg, 95%) (all % throughout the experimental section or main text refer to yields unless specified otherwise) (Scheme1). The purity of the compound was >90% judged from the NMR data.

**Figure 1:**
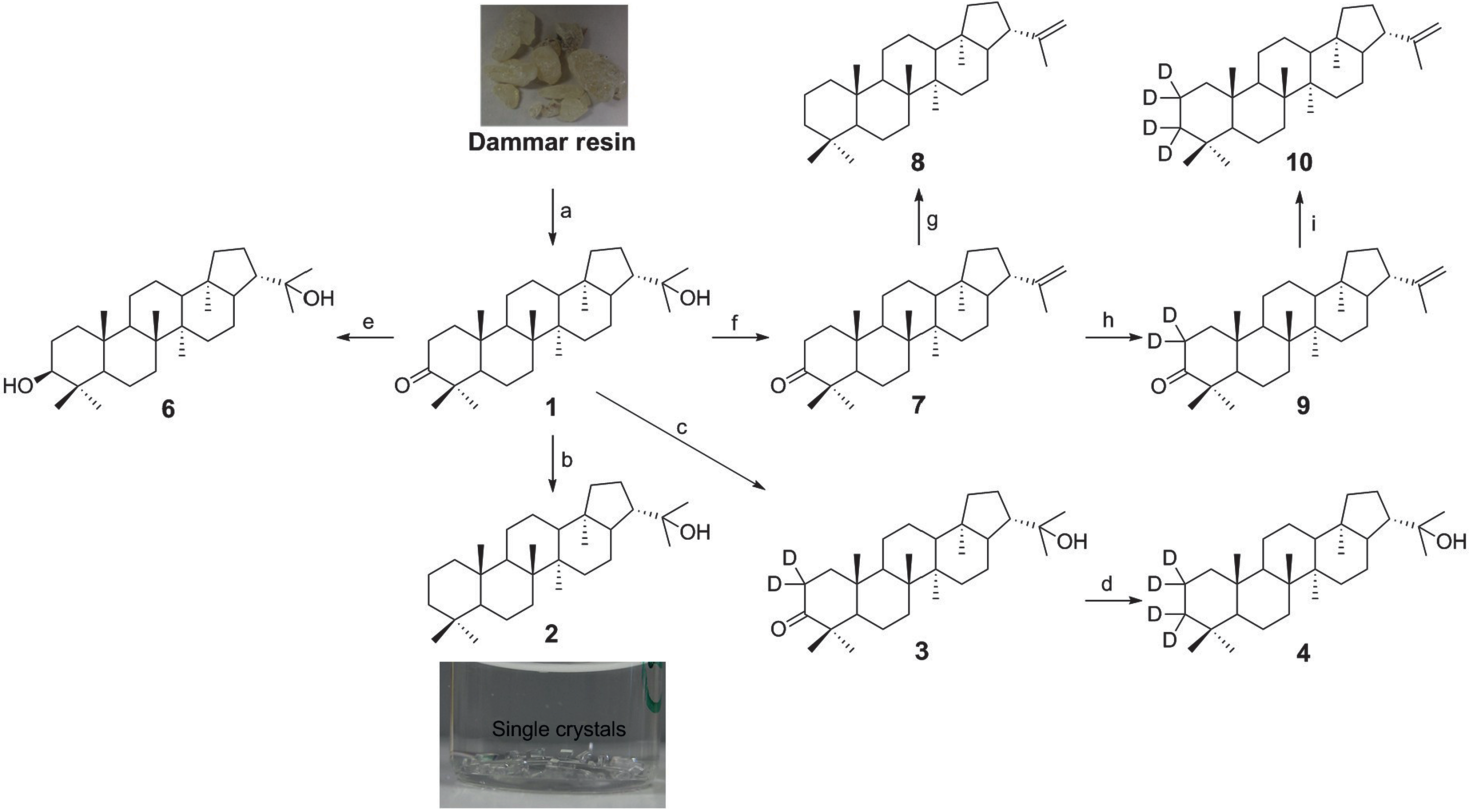
Scheme Synthesis of hopanoid derivatives from hydroxyhopanone (1). Numbers represent different hopanoids shown in Fig.2. Reagents and conditions: (a) extraction, 0.77%; (b) i. NH_2_NH_2_, DEG, reflux; ii. NaOH, 200–225 ^o^C, 95%; (c) NaOD, D_2_O-MeOD-CD_2_Cl_2_; (d) CH_3_C_6_H_4_SO_2_-NHNH_2_, NaBD_4_, D_6_-toluene-MeOD, 44% in two steps; (e) NaBH_4_, MeOH-Et_2_O, 90%; (f) PdCl_2_, anhydrous toluene, MS 3Å, 70 °C, 40%; (g) NaBH_4_, anhydrous toluene-MeOH, MS 3Å, reflux, 82%; (h) NaOD, CD_2_Cl_2_-MeOD-D_2_O, 100%; (i) NaBD_4_, anhydrous toluene-MeOD, MS 3Å, reflux, 90%.

To obtain 2,2-D_2_-hydroxyhopanone (**3**, Fig.2), purified hydroxyhopanone (250 mg, 0.57 mmol) was dissolved in CD_2_Cl_2_ and MeOD in a glass flask. NaOD in D_2_O (40 wt. %) was added. The reaction was stirred overnight and then quenched by pumping a dry carbon dioxide flow into the reaction mixture until the pH is ∼7. The deuterated product was extracted by an ether/water extraction. The organic fraction was dried over magnesium sulfate and filtered. The solvent was removed using a rotary evaporator to afford 2,2-D_2_-hydroxyhopanone (3) as colorless crystals in 100% yield (250 mg) (Scheme1).

To obtain 2,2,3,3-D_4_-diplopterol (4), the purified 2,2-D_2_-hydroxyhopanone (3) (145 mg, 0.33 mmol) was dissolved in a mixture of MeOD and D_6_-toluene (10 + 20 mL). 4-Toluenesulfonyl hydrazide (303 mg, 1.63 mmol) was added to the solution, followed by heating under reflux with a Soxhlet condenser filled with activated 3Å molecular sieves. After 4 h, the solution was cooled to RT and NaBD_4_ (27 mg, 652 μmol) was added. After heating under reflux for 2 h, the solution was cooled to RT and concentrated. The residual material was subjected to silica chromatography (petroleum: diethyl ether = 11:2) to afford the 2,2,3,3-D_4_-diplopterol a colorless solid (62 mg, 44%) (Scheme1; Fig. 7C).

To obtain 2,2,3,3-D_4_-diploptene (10), 2,2-D_2_-3-oxo-diploptene (9) was prepared, followed by enolization and reduction. Briefly, PdCl_2_ (400 mg, 2.3 mmol) was added to the solution of hydroxyhopanone (2.0 g, 4.7 mmol) in anhydrous toluene (200 mL)-CH_2_Cl_2_ (10 mL) with 3 Å molecular sieves. The reaction was kept under an atmosphere of Ar at 70 °C for 5 h. The resulting suspension was filtered through Celite, and the filtrate was concentrated. The residual was subjected to Ag-coated silica chromatography (Morris, 1966) (hexane:Et_2_O = 20:1) to afford the desired product 3-oxo-diploptene (7) (0.8 g, 40%), which was further crystallized in CH_2_Cl_2_-hexane for later use. **7** (100 mg, 0.24 mmol) was dissolved in CD_2_Cl_2_ (5.0 mL)-MeOD (1.67 mL)-D_2_O (0.25 mL) in a glass flask. NaOD in D_2_O (40 wt. %, 13 μL) was added. The reaction was stirred overnight and then quenched by pumping a dry carbon dioxide flow into the reaction mixture until the pH is ∼7. The deuterated product was extracted using an ether/water extraction. The organic fraction was dried over magnesium sulfate and filtered. The solvent was removed using a rotary evaporator to afford product **9** as colorless crystals in 100% yield (100 mg) (Scheme1). **9** (50 mg, 0.12 mmol) was dissolved in toluene (20 mL). 4-Toluenesulfonyl hydrazide (44 mg, 0.23 mmol) was added to the solution, followed by heating under reflux with a Soxhlet condenser filled with activated 3 Å molecular sieves. After 2 h, the solution was cooled to RT. NaBD_4_ (20 mg, 0.48 mmol) and D_4_-methanol (5 mL) were added in two portions over 10 min. After heating under reflux for another 30 min, the solution was cooled to RT and concentrated. The residual material was subjected to silica chromatography (hexane) to afford 2,2,3,3-D_4_-diploptene, a colorless solid (45 mg, 90%) that was further crystalized as small needles in hexane-CH_2_Cl_2_ (Scheme1; Fig. 7D).

To obtain diploptene, **7** (50 mg, 0.12 mmol) was dissolved in toluene (20 mL). 4-Toluenesulfonyl hydrazide (42 mg, 0.22 mmol) was added to the solution, followed by heating under reflux with a Soxhlet condenser filled with activated 3 Å molecular sieves. After 2 h, the solution was cooled to RT. NaBH_4_ (20 mg, 0.53 mmol) and methanol (5 mL) were added in two portions over 10 min. After heating under reflux for another 30 min, the solution was cooled to RT and concentrated. The residual material was subjected to silica chromatography (hexane) to afford diploptene, a colorless solid (41 mg, 82%) that was further crystallized as small needles in hexane-CH_2_Cl_2_ (Scheme1; Fig. 7B).

### X-ray diffraction, data collection, and structure solution

Low temperature (Cosier & Glazer, 1986) single crystal X-ray diffraction was collected for **2** and **11** using an Oxford Diffraction (Agilent, Cheshire, UK) Supernova A diffractometer and a Nonius KappaCCD diffractometer, respectively. Raw frame data were collected and reduced using CrysAlysPro or DENZO-SMN/HKL-COLLECT (Otwinowski & Minor, 1997) as appropriate. Structure solution was carried out using charge flipping (Oszlanyi & Suto, 2004) within SuperFlip (Palatinus & Chapuis, 2007), and the structures were refined using CRYSTALS (Betteridge *et al*., 2003). On initial refinement of the structure of **2**, the displacement ellipsoids were poorly shaped and R1 persisted at 18%. Examination of the Fo/Fc plot suggested the presence a second component and examination of the cell parameters suggested this might involve twinning by rotation about the (-1 0-1) direction. On inclusion of a suitable twin law (Cooper *et al*., 2002), R1 dropped to <8% and the shape of the displacement ellipsoids improved. In addition to the twinning described above, as the structure is non-centrosymmetric, it was also necessary to consider the possibility of the primary component of the crystal being itself a racemic twin (‘conglomerate’). An attempt was made to treat the crystal as a four-component twin, and refinement of the relative contributions of the four components to the diffraction pattern gave values of ca. 0.6, 0.4, 0, and 0, respectively. It was therefore concluded that the crystal is twinned purely by rotation and that each component is a single enantiomer. For **11**, FRIEDIF (Flack & Shmueli, 2007) was found to be 3.48, well below the recommended 80 for absolute configuration determination for enantiopure materials (Thompson & Watkin, 2011). All non-hydrogen atoms were refined using anisotropic displacement ellipsoids, and hydrogen atoms were refined with restraints prior to inclusion in the final refinement with a riding model (Cooper *et al*., 2010). Full refinement details are given in the Supporting information (CIF). Crystallographic data (excluding structure factors) have been deposited with the Cambridge Crystallographic Data Centre, and copies of these data can be obtained free of charge *via* www.ccdc.cam.ac.uk/data_request/cif (1022080 for 2; 1022081 for 11).

## Results and Discussion

### Genetically tractable *R. palustris* TIE-1 as a tool for microbial production of hopanoids

Earlier studies of hopanoids isolated from bacteria were mostly in trace amounts sufficient for NMR and MS characterization. Purification of hopanoids such as BHT (13), BHT glucosamine (16), and BHT cyclitol ether (17) in larger quantities (mg) as calibration standards for LC has been reported using an obligate ethanologenic bacterium *Zymomonas mobilis* (Schulenberg-Schell *et al*., 1989; Moreau *et al*., 1995). However, *Z. mobilis* does not contain *hpnP*, the methylase gene required for 2-methylhopanoid production. Here, we optimized methylated hopanoid production and purification using the model hopanoid-producing organism *R. palustris* TIE-1. Our knowledge of the hopanoid biosynthetic pathway in this organism and the existence of diverse hopanoid mutant strains and cloning vectors facilitated these efforts (Welander *et al*., 2012). For example, using an IPTG-inducible *hpnP* and *hpnO* (encoding a transaminase) enabled us to increase the yield of 2-methylhopanoids and aminobacteriohopanetriol (aminotriol), respectively. Use of an *hpnPO* knockout strain allowed purification of BHT without a need to separate it from 2Me-BHT and aminotriol. Similarly, the production of C_30_ hopanoids and adenosylhopane was enhanced using the Δ*hpnH* and Δ*hpnG* strains, respectively, in which the genes responsible for the production of extended (C_35_) hopanoids are missing (Welander *et al*., 2012).

### Optimization of 2-methylhopanoid production in *R. palustris* TIE-1

In addition to using mutant strains, we further optimized the production of 2-methylhopanoids by manipulating the growth conditions. Two strains, *R. palustris* TIE-1 WT and DKN1283, were selected for a high throughput screen to optimize growth in 96-well plates. DKN1283 has an extra copy of the hopanoid 2-methylase *hpnP* inserted into the genome under an IPTG-inducible promoter. To the standard medium YPMS, squalene, the biosynthetic precursor of hopanoids (Seckler & Poralla, 1986), methionine, the methyl donor in cells (Zundel & Rohmer, 1985; Rashby *et al*., 2007), or vitamin B12, a cofactor for the 2-methylase HpnP was added (Welander *et al*., 2010). Effects from the addition of acetate as carbon source or the removal of MOPS and succinate from the medium was also examined. The growth curves of WT and DKN1283 in the absence or presence of light are shown in Fig. S1. No significant difference between WT and DKN1283 (with IPTG induction) was observed. The addition of squalene, methionine, or vitamin B12 did not affect the final density of the cells. Interestingly, the addition of acetate resulted in 50–100% increase in the final OD_600_ compared to YPMS only, and the phototrophic condition was able to support higher cell density (∼2×).

Next, the hopanoid contents in these cells were examined. Protocols to extract all of the lipids (total lipid extract) from small volumes of cell culture (<1 mL) were developed, and their comparable extraction efficacy as protocols for extraction from large-scale cultures (>300 mL culture) was verified by GC-MS. The total lipid extracts were acetylated, and the quantification of diplopterol (2), BHT (13), and their respective 2-methylated species was analyzed by GC-MS. The results are summarized in Table S3. The culture grown in YP did not yield sufficient cell mass for extraction and was omitted from lipid analysis. Because purified hopanoid standards were not available at the time, assumption of equal ionization efficiency between diplopterol and BHT was made to allow quantification of these hopanoids. Although this assumption was subsequently found to be inaccurate, our conclusion about which growth condition allows maximal 2Me-BHT (15) production still holds.

The additives squalene, vitamin B12, or methionine did not increase the level of 2-methylation, suggesting that despite their roles in hopanoid biosynthesis and methylation, these molecules are not the limiting factors in the production of 2-methylhopanoids under the conditions tested. In contrast, the addition of acetate greatly enhanced the production of 2-methylhopanoids (Table S3). As *R. palustris* TIE-1 utilizes the non-mevalonate pathway without involving acetyl-CoA for isoprenoid synthesis, the mechanism through which acetate increases the 2-methylhopanoid synthesis is unclear (Rohmer *et al*., 1993). Compared to C_30_ hopanoids, the C_35_ hopanoids showed a lower level of 2-methylation, both in WT and DKN1283, regardless of the presence or absence of light. Among all the conditions, DKN1283 grown in YPMS with acetate and light had the highest percentage of 2Me-BHT (10% of total hopanoids) and highest yield of 2Me-BHT per volume of culture. Accordingly, we used this growth condition to optimize 2Me-BHT production on a larger scale.

To scale up the production of 2Me-BHT, multiple 2-L flasks were used to increase the culture volume. Different amounts of MOPS, succinate, and acetate were tested, and methionine was also included as a control. Fig. S2 (A,B) compares the percentage of 2Me-BHT in total hopanoids and the ratio of 2Me-BHT (15) to BHT (13) among these growth conditions. Similar to the small-scale growth screen, the addition of methionine did not increase 2Me-BHT production. Decreasing amounts of MOPS resulted in lower 2Me-BHT production, whereas increasing the concentration of the carbon source (succinate or acetate) enhanced the ratio and percentage of 2Me-BHT. Interestingly, the 2Me-BHT to BHT ratio reached 1.75 at 20 mm acetate, which is 3.3-fold higher compared to the optimum condition in the small-scale screen (2Me-BHT/BHT ∼0.53).

Encouraged by these results, different combinations of MOPS, succinate, and acetate were tested (Fig. S2C,D). Some improvement of the 2Me-BHT production using 20 mm succinate and 40 mm acetate was observed and time course studies indicated that the 2Me-BHT to BHT ratio increased over time during late stationary phase and doubled to over 1.3 on the 7th day, compared to 0.6 at the beginning of stationary phase (Fig. S2E). Under these conditions, the 2Me-BHT/BHT ratio and percentage of 2Me-BHT increased over 25- and 12-fold, respectively, compared to the original YPMS-only condition before screening. We therefore chose these conditions to prepare cells for total lipid extraction and hopanoid purification.

### Purification of hopanoids by normal- and reverse-phase chromatography

**Figure 3:**
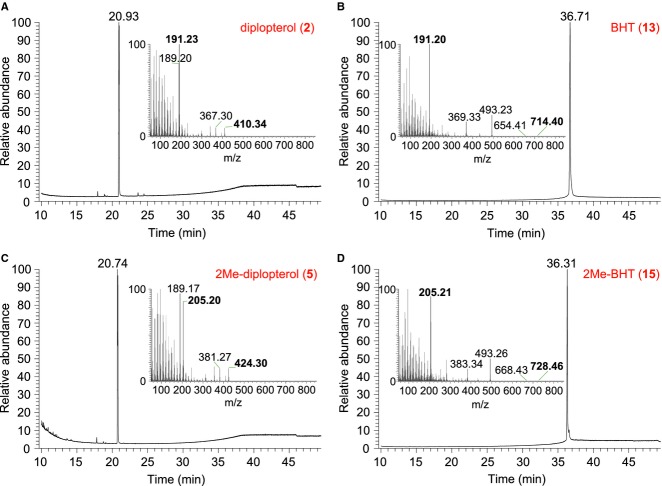
GC-MS of purified hopanoids from *R. palustris* TIE-1. Purified hopanoids were acetylated and analyzed by GC-MS. (A) diplopterol (2), (B) BHT (13), (C) 2Me-diplopterol (5), (D) 2Me-BHT (15). Inserts: MS spectra of each purified hopanoid with characteristic ion m/z ratios highlighted in bold.

**Figure 4:**
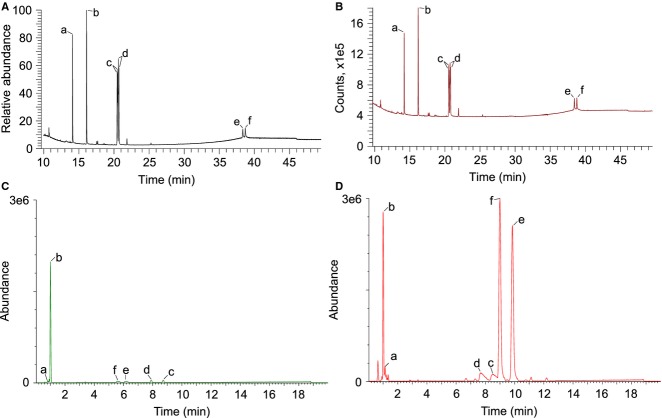
GC-MS/FID and LC-MS of hopanoid and sterol standards. 7 ng of acetylated samples was analyzed by GC-MS (A) or GC-FID (B), and 50 ng of non-acetylated (C) or acetylated (D) samples was analyzed by UPLC-ESI-TOF-MS. a: androsterone, b: pregnane acetate, c: 2Me-diplopterol (5), d: diplopterol (2), e: 2Me-BHT (15), f: BHT (13) (note: different unit in *Y*-axis reflects different detection methods).

Efforts to improve the purification of hopanoids are described in detail in the Supporting Information. Table1 summarizes the yields in each purification step from three 48 L cultures. An average of ∼72 g of wet cell paste was obtained, which is the equivalent of ∼24 g of dry cell weight after lyophilization. The yield of total lipid extract (TLE) was ∼14% w/w of dry cells. The average final yields of diplopterol, 2Me-diplopterol, BHT, and 2Me-BHT were 3 mg, 53 mg, 11 mg, and 24 mg, respectively, which in combination represent ∼3% w/w of TLE. As each purification step incurred some loss, and because some hopanoids were not isolated in our methods, the real percentage of total hopanoids in lipid extracts is higher than 3%. The yield of 2Me-diplopterol and 2Me-BHT was ∼12–34× and 2× higher than their respective unmethylated hopanoids. Diplopterol and 2Me-diplopterol ((2Me)-diplopterol) and BHT and 2Me-BHT ((2Me)-BHT) purity were assessed by GC-MS, and a dominant single peak was observed for each hopanoid (Fig.3). The m/z of (2Me)-diplopterol in the MS spectra indicates that dehydration of the tertiary alcohol occurred in the GC column. These purified hopanoids were also analyzed by LC-MS (Fig. S3). Similar to GC-MS, (2Me)-diplopterol was dehydrated, regardless of acetylation or not. Even though non-acetylated hopanoids can be readily detected in our UPLC-ESI-TOF-MS, the signals were much weaker than the acetylated samples (Fig.4).

**Table 1:** Summary of purification of hopanoids from *R. palustris* TIE-1

			Silica gel (mg)			HPLC (mg)				De-acetylation (mg)	
	Cell paste (g)	TLE (g)	Diploptene(8)	(2Me)-Dip(5)	(2Me)-BHT-4Ac	2Me-Dip(5)	Dip(2)	2Me-BHT-4Ac	BHT-4Ac(14)	2Me-BHT(15)	BHT(13)
Batch 1	79.5	4.0	45.0	437.7	72.5	62.2	5.2	41.2	9.9	33.3	7.8
Batch 2	63.0	3.5	28.5	180.2	40.5	47.0	1.4	16.4	15.2	13.8	11.5
Batch 3	72.2	2.4	294.9	275.3	76.7	47.1	2.6	30.1	15.7	25.6	13.8
Average	71.6	3.3	122.8	297.7	63.2	52.8	3.1	29.2	13.6	24.2	11.0
SD	8.3	0.8	149.3	130.2	19.8	9.9	1.9	12.4	3.2	9.8	3.0

Ac, acetylation; (2Me)-hopanoid, hopanoid and 2Me-hopanoid; SD, standard deviation.

### Use of purified hopanoids as MS standards

It is well known that diverse hopanoids have different signal intensities on either GC-MS or LC-MS, and therefore, calibration using purified hopanoids in parallel with sterol internal standards is typically used for quantification. For example, detailed comparisons of signal intensities from APCI-LC-MS between acetylated hopanoids and 5-α-pregnane-3β, 20β-diol have been reported (Cooke, 2011). Four hopanoids with one or more nitrogen atoms including aminotriol, adenosylhopane, BHT glucosamine, and BHT cyclitol ether have, on average, 12× stronger signal than the internal standard, whereas BHT has an 8× stronger signal than the standard (van Winden *et al*., 2012). These response factors depend on individual instrument parameters and the types of detectors, but even after controlling for these factors, there is still about 20% variation in signal intensities from runs on different days, possibly due to fluctuations in room temperature or humidity (Cooke, 2011).

**Figure 5:**
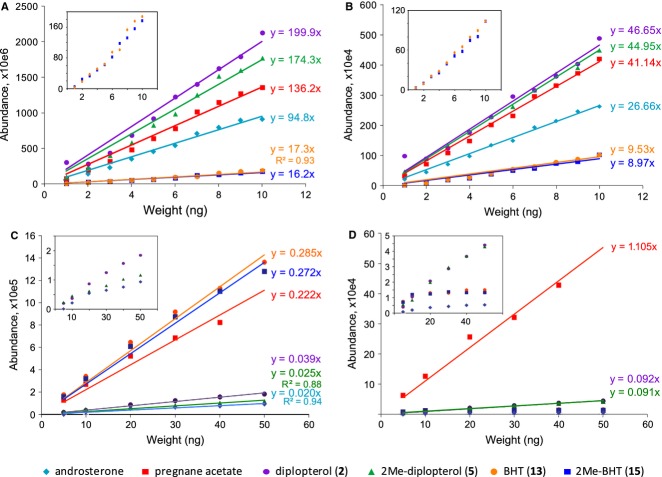
Calibration curves for various signal responses between purified hopanoids and sterol standards in GC-MS/FID and LC-MS. (A) GC-MS, (B) GC-FID, (C) LC-MS, acetylated samples, (D) LC-MS, unacetylated samples. Examples from three replicates are shown. Equations indicate the results of each linear fit, and all R-square values are between 0.95 and 0.99, except those stated in the figure. Inserts: *y*-axis blown-up to show samples that have low signal intensities (some samples do not have linear signal response and therefore were not fitted (D)).

To examine the difference in ionization efficiencies among the common standards androsterone and pregnane acetate, and purified (2Me)-diplopterol and (2Me)-BHT, we analyzed various amounts of samples (Table2, Fig.5). When analyzed by GC-MS, methylation at the 2′-position does not affect the ionization efficiency significantly. Furthermore, (2Me)-BHT has only ∼0.1× of the signal of (2Me)-diplopterol. Compared to androsterone, (2Me)-diplopterol and (2Me)-BHT showed ∼2× and 0.18× signal intensity, respectively. These results indicate that use of androsterone as a standard, assuming equal ionization efficiencies, systematically overestimates and underestimates the amounts of (2Me)-diplopterol and (2Me)-BHT, respectively. Interestingly, when using a flame ionization detector (FID) after the GC column, in which the signal is expected to be proportional to the amount of carbon in the sample, the relative signal intensities show dramatic deviation from their total mass. (2Me)-diplopterol still has ∼5× stronger signal than (2Me)-BHT and ∼1.7× higher signal than androsterone. These results suggest that the structural diversity of hopanoids can not only affect the signal intensity in the MS detector, but also affect ‘structure-independent’ detection methods such as FID.

**Table 2:** Signal response ratios between same amounts of hopanoids and sterol standards in GC-MS/FID and LC-MS. Standard deviation from three replicates are shown

	Androsterone	Pregnane acetate	2Me-Dip (5)	Dip (2)	2Me-BHT (15)	BHT (13)
GC-MS	1	1.45 ± 0.01	1.83 ± 0.04	2.10 ± 0.04	0.18 ± 0.02	0.18 ± 0.02
GC-FID	1	1.53 ± 0.01	1.69 ± 0.01	1.73 ± 0.01	0.35 ± 0.02	0.37 ± 0.02
LC-MS	0.088 ± 0.001	1	0.111 ± 0.003	0.168 ± 0.005	1.202 ± 0.019	1.270 ± 0.014
LC-MS*		1	0.079 ± 0.003	0.081 ± 0.002		

* Non-acetylated androsterone and hopanoids; androsterone, 2Me-BHT, and BHT have nonlinear response.

Similar analyses were carried out in LC-MS (Table2). Acetylated samples show a much better linear relationship between amounts of material and corresponding signal intensities, even though the signals from androsterone and (2Me)-diplopterol are much weaker than the rest (Fig.5C). (2Me)-BHT shows ∼8–11× and 1.2× signal than (2Me)-diplopterol and pregnane acetate, respectively. Non-acetylated hopanoids can also be detected in LC-MS (Fig.5D). However, no strong linear response can be observed for androsterone and (2Me)-BHT, probably due to low solubility of these compounds in the LC-MS solvent.

Several factors could contribute to the variation in signal intensities of (2Me)-BHT and (2Me)-diplopterol in the different detection methods. The first is the inherent lower ionization efficiency of the molecules in GC-MS or LC-MS. In GC-MS, the lower signal could be due to sample loss at the injection line or loss at higher temperature from leakages in connectors. The first factor can be reliably calibrated by external standards using purified hopanoids; however, the latter two are less predictable and can fluctuate dramatically between samples analyzed on different days. There are two possible strategies to address such instrumentation variability. The first would be to run external standards before and after each analysis, but this has the disadvantages of requiring longer instrument time and increased mobile phase consumption. Alternatively, proper deuterated hopanoids, that is, hopanoids incorporated with enough deuterium atoms to introduce a mass shift high enough to avoid overlap of isotope envelopes of labeled and unlabeled hopanoids, could be included in the samples as internal standards to account for variations in instrument conditions.

### Synthesis of 2,2,3,3-D_4_-diplopterol (4) and 2,2,3,3-D_4_-diplotene (10)

Previously, two approaches were employed to obtain deuterated hopanoids (Pan & Vincent, 2008; Doughty *et al*., 2014). One approach was to grow cells in the presence of deuterated water, and purify the labeled hopanoids (Doughty *et al*., 2014). However, the yield from this method was very low and the final products contained mixtures of deuterated and non-deuterated hopanoids, making it unsuitable for internal standard calibration. The other approach involved the synthesis of BHT from hydroxyhopanone extracted from Dammar resin, followed by attachment of dideuterated glucosamine to BHT (Pan & Vincent, 2008). The resulting compound is a good internal standard for calibrating nitrogen-containing hopanoids containing, but it is not suitable for (2Me)-diplopterol and (2Me)-BHT. Here, we developed a novel method to introduce deuterium at the hopanoid pentacyclic backbone that can be retained during the downstream synthesis of more complex hopanoid standards, including BHT, BHT-glucosamine (16), and BHT-cyclitol ether (17). Furthermore, our approach produced tetradeuterated hopanoids, which have MS signals further separated from the neutral hopanoids compared to the dideuterated standards, providing advantages for easier data analyses. The synthesized tetradeuterated hopanoids can then be further converted to N-containing hopanoids such as BHT-glucosamine by published protocols (Pan *et al*., 2005, 2007).

**Figure 6:**
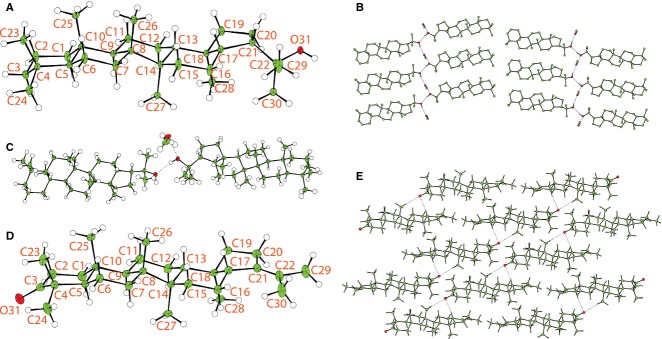
The crystal and molecular structures of diplopterol (2) and 3-oxo-21-enedeoxyhopane (11). (A) Displacement ellipsoid plot of 2 showing the stereochemistry, with the ADPs shown at 30% probability and the hydrogen atoms shown with arbitrary radius. The second molecule is numbered as the first with 50 added to the serial number. (B) Packing diagram of 2 showing the O-H…O hydrogen-bonded chains (hydrogen atoms bound to carbon omitted for clarity). Hydrogen bond distances are as follows: O31-H311…O81: 2.756(3) Å; O81-H811…O92: 2.765(3) Å; O92-H921…O31: 2.722(3) Å. (C) Asymmetric unit for 2 with ADPs drawn at 50% probability showing the hydrogen bonds as dotted lines. (D) Displacement ellipsoid plot of 11 showing the asymmetric unit and stereochemistry, with the ADPs shown at 30% probability and the hydrogen atoms shown with arbitrary radius. (E) Packing diagram of 11 showing the weak C-H…O interactions as a dotted line. C-H…O distances are C17-H171…O3: 3.569(3) Å and C26-H261…O3: 3.419(3) Å.

**Figure 7:**
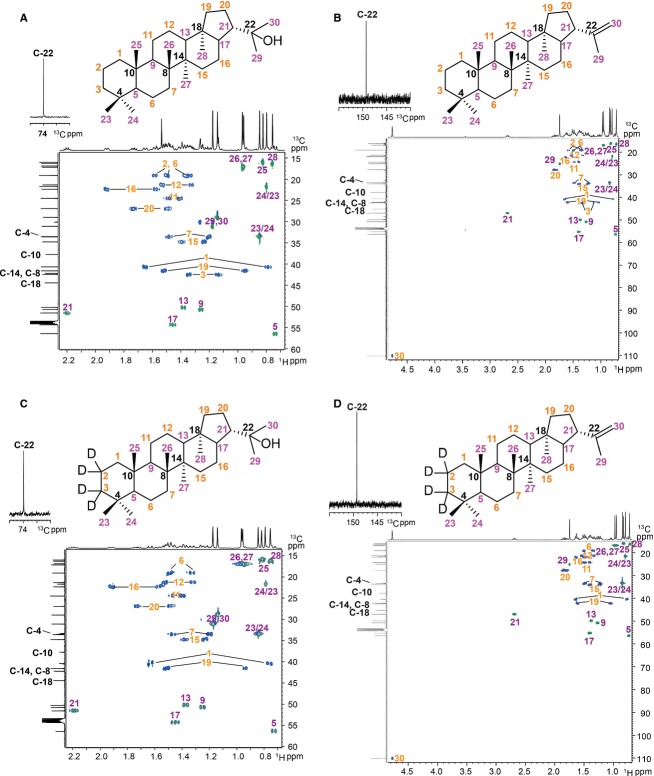
2D NMR spectra of synthetic hopanoids. (A) diplopterol (2), (B) diploptene (8), (C) 2,2,3,3-D_4_-diplopterol (4), (D) 2,2,3,3-D_4_-diploptene (10). CH_3_ and CH groups are highlighted with purple numbers, while CH_2_ groups with orange numbers and quaternary carbons with black numbers.

To synthesize the deuterated hopanoids, we selected hydroxyhopanone as a starting material, because gram quantities of this hopanoid can be extracted from inexpensive and readily available Dammar resin. Furthermore, the ketone at the 3′-position of hydroxyhopanone provides a synthetic handle to introduce deuterium into 2′- and 3′-positions to produce 2,2,3,3-D_4_-diplopterol. The reported isolation of hydroxyhopanone from the plant-derived Dammar resin gave a low yield (0.34%). However, a modification of the extraction protocol resulted in the yield doubling (Supporting information). To examine whether the stereochemistry of hopanoids isolated from Dammar resin and *R. palustris* TIE-1 is identical, Wolff–Kishner reduction of hydroxyhopanone to diplopterol was carried out with a yield of 95% yield. Single crystals of diplopterol were obtained, and its crystal and molecular structure was determined (Fig.6). NMR data from diplopterol purified from *R. palustris* TIE-1 and that from Dammar resin are identical, suggesting no structural variation between material from the two sources (Fig.7A). Diplopterol was found to crystallize in the monoclinic space group P2(1) with two diplopterol molecules and one molecule of methanol in the asymmetric unit. The structure forms strong O-H···O D_2_^2^(4) hydrogen-bonded chains connecting the diplopterol molecules and methanol solvent. These are connected through hydrophilic interactions to form a bilayer structure (Fig.6B). In contrast, **11** formed a layered structure with weak C-H···O interactions within the sheets (Fig.6D, E). The absolute and relative stereochemistry of diplopterol isolated from Dammar resin was consistent with those proposed previously in the literature (Corbett & Smith, 1967; Tsuda *et al*., 1967).

To synthesize 2,2,3,3-D_4_-diplopterol, enolization of **1**, using NaOD in a mixture of D_2_O, MeOD, and CD_2_Cl_2_, gave **3** with deuterium incorporated at the 2-position, in 100% yield (Scheme1). However, trials to reduce **3** to **4** under the same condition as that for diplopterol, while replacing NaOH with NaOD and diethylene with D_2_-diethylene, did not give any conversion to the desired product **4**. An increase in deuterium incorporation was made by treatment of **3** in toluene and MeOD with 4-toluenesulfonyl hydrazide, followed by reduction with sodium borodeuteride (NaBD_4_) to produce the desired tetradeuterated diplopterol **4** in 44% yield over two steps (Scheme1; Fig.7).

The tertiary alcohol of 2,2,3,3-D_4_-diplopterol is susceptible to elimination under the conditions found in a mass spectrometer. However, 2,2,3,3-D_4_-diploptene instead is much more stable in biological environments and under conditions required for mass spectrometry. This compound can also be elaborated to give 2,2,3,3-D_4_-BHT and 2,2,3,3-D_4_-BHT-glucosamine (Pan *et al*., 2005). We therefore sought to synthesize 2,2,3,3-D_4_-diploptene. Direct elimination of the tertiary alcohol of diplopterol (2) with phosphoryl chloride (Dunstan *et al*., 1957; Duvold & Rohmer, 1999) resulted in a mixture of diploptene and other isomers that were inseparable. An alternative approach is to synthesize 3-oxo-diplotene (7), followed by deuterium incorporation and subsequent reduction of ketone, as in the preparation of **4**. We undertook reaction optimization to give a higher yield of the desired isomer **7**. After evaluating 18 conditions using 12 Lewis acids (Fig. S4), PdCl_2_ was found to be the most effective Lewis acid, giving 40% of **7** (isolated yield) when the reaction was carried out at 70 **°**C. Silver-coated silica chromatography was used to separate **7** from other isomers. Subsequent formation of the hydrazone with toluenesulfonyl hydrazide, followed by reduction with sodium borohydride, gave diploptene in 82% yield. Conversion of **7** to **9** in deuterium oxide in the presence of sodium deuteroxide was successful in 100% yield. Subsequent formation of hydrazone with toluenesulfonyl hydrazide followed by reduction with sodium borodeuteride gave 2,2,3,3-D_4_-diploptene **(10)** in 90% yield (Scheme1; Fig.7).

The field ionization (FI) MS measurements revealed that **4** is a mixture of D_2_-, D_3_-, and D_4_-diplopterol with the ratio of 6%: 37%: 57%, while **10** is a mixture of D_2_-, D_3_-, and D_4_-diploptene with the ratio of 4%: 31%: 65%. The incomplete incorporation of four deuterium atoms is mainly due to the varying levels of deuterium in the reagents and potentially the lower reactivity of deuterated reagents compared to their undeuterated equivalents (Lu *et al*., 2010).

### Deuterated hopanoids as internal standards

**Figure 8:**
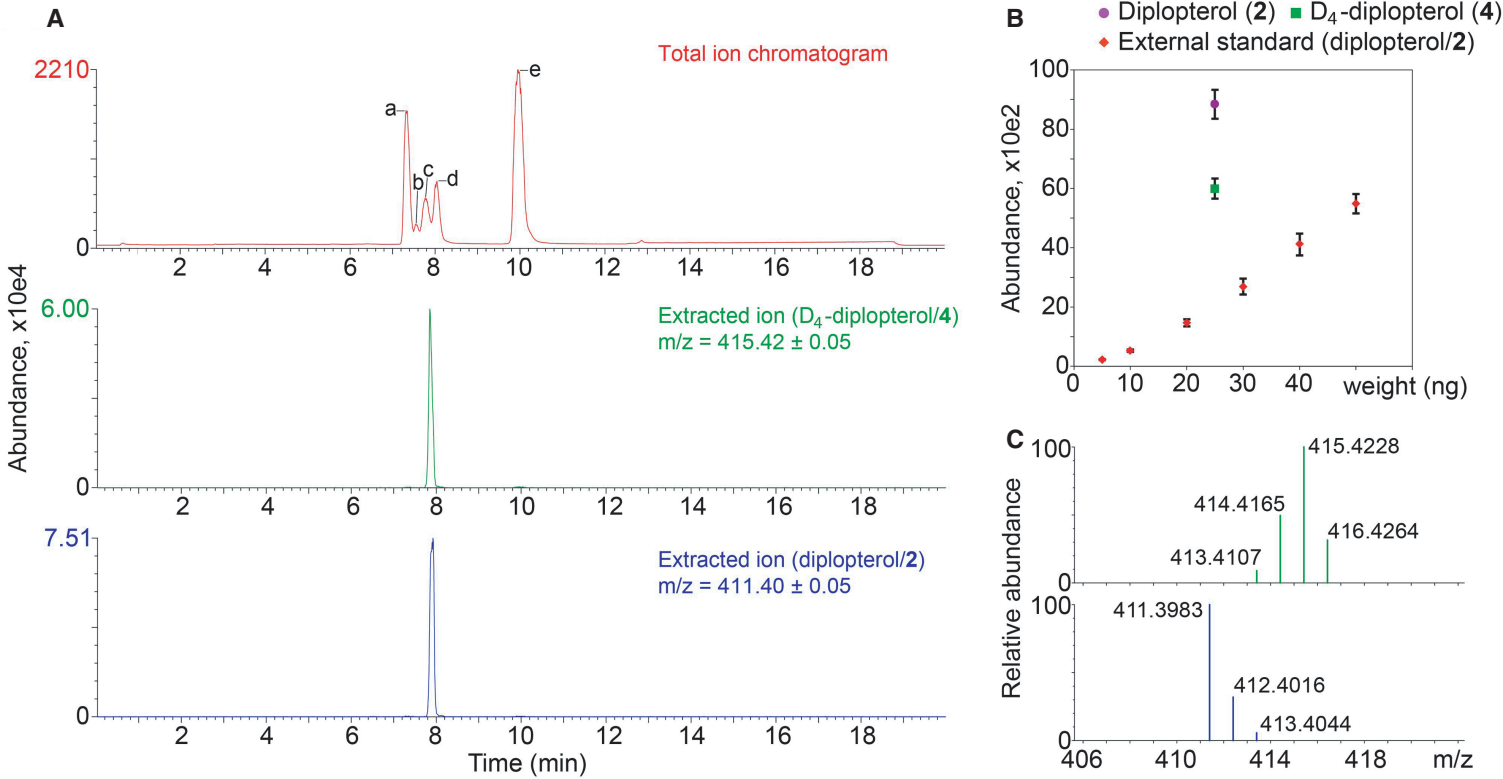
Use of D_4_-diplopterol (4) as an internal standard for diplopterol (2) in LC-MS. Equal amounts (25 ng) of unacetylated D_4_-diplopterol and diplopterol were mixed with 250 ng each of a: PC (16:0/16:0), b: PS (17:0/17:0), c: PG (17:0/17:0), d: PG (18:0/18:1), and e: PC (18:0/18:1). (A) LC-MS total ion chromatogram of the mixture, and the extracted ions that represent D_4_-diplopterol (m/z 415.4) and diplopterol (m/z 411.4) are shown. *Y*-axis shows the absolute value of signal intensity from MS. (B) Comparison between signal responses of diplopterol (m/z 411.4) and D_4_-diplopterol (m/z 415.4) in the mixture and an external standard of purified diplopterol (m/z 411.4). Error bars represent standard deviation from three technical replicates. (C) Mass spectra of individual D_4_-diplopterol (upper) and diplopterol (lower).

As a proof of concept, to test the efficacy of synthetic deuterated hopanoids as internal standards for quantification, equal amounts of diplopterol (2) and D_4_-diplopterol (4) (25 ng) were mixed with 250 ng each of different phospholipids and analyzed by LC-MS (Fig.8). The total ion chromatogram shows that phosphatidylglycerol (PG) (17:0/17:0) co-elutes with (D_4_)-diplopterol. To examine the impact of co-elution on the ionization efficiency of diplopterol, the signal intensity representative of diplopterol (m/z 411.4) was compared to those of purified diplopterol external standard (Fig.8B). Using the linear fitting curve of the external standard, the amount of diplopterol in the phospholipid mixture is 80.0 ng, which is 220% higher than the real value (25 ng). This result is opposite of what we would expect because ion suppression by co-eluted molecules leads to lower signal intensity. However, in this specific case, it appears that PG (17:0/17:0) enhances the ionization efficiency of diplopterol. Contrary to the external standards, the signal intensity of D_4_-diplopterol is similar to diplopterol (Fig.8B). The MS profile of D_4_-diplopterol in the LC-MS indicates a mixture of 60.3%, 33.3%, and 6.4% of D_4_-, D_3_-, and D_2_-diplopterol (Fig.8C). Therefore, 84.6% of the m/z 415.4 signal comes from D_4_-diplopterol. Adjusting the purity of D_4_-diplopterol and the m/z 415.4 signal from D_4_-diplopterol, the calculated amount of diplopterol from D_4_-diplopterol is 26.3 ng, which is much closer to the real value compared to one from the external standard. This example shows that, in a complex mixture not resolvable by chromatography, the ionization efficiency of hopanoids can be greatly affected by other co-eluted compounds, and an internal deuterated hopanoid standard can account for such effects and enable more accurate hopanoid quantification.

### Geobiological implications

Geobiology is a discipline that requires comparative analyses to make sense of complexity: pattern detection and correlations are the starting point for hypotheses regarding what set of factors (biological, chemical, physical) drive modern or ancient processes. Charting the occurrence of specific hopanoids, be it within cellular membranes or ancient rocks, has been used to detect hopanoid distribution patterns that inform inferences about their biological function(s) and/or their evolutionary significance. For example, the enrichment of 2Me-hopanoids in the outer membrane of the akinete cell type of *Nostoc punctiforme* pointed to a role for 2Me-hopanoids in stress resistance (Doughty *et al*., 2009); peaks in the 2Me-hopane index during the Phanerozoic correlate with episodes of ocean anoxia, possibly reflecting a link between hopanoids and nitrogen fixation (Knoll *et al*., 2007). These types of correlations pave the way for other studies to examine causality, but to justify such efforts, it is vital that distribution patterns be robust and comparable for similar environments. Toward this end, accurate and reproducible hopanoid analyses between laboratories and studies are essential. Just as awareness of the potential for contamination of ancient samples has led to re-evaluation of the antiquity of 2Me-hopanes (Rasmussen *et al*., 2008; Brocks, 2011), recognition of potential analytical biases in hopanoid quantification will help different laboratories standardize methods and compare results. This has the potential to enable meaningful metadata sets to be collected, permitting better identification of reliable indicators for particular environmental processes. Without precise and accurate concentrations of hopanoids/hopanes, those interested in understanding the distribution and meaning of these molecules in modern or ancient environments run the risk of missing important patterns because values from different studies may be offset by unknown errors. For instance, without using proper hopanoid standards, it would be easy to overestimate the relative abundance of short hopanoids (C_30_) vs. long hopanoids (C_35_) in a sample. Extending the type of approach described in this study to other hopanoid compounds will improve absolute and accurate values to characterize their distribution within cells and the larger environment. A formidable but worthwhile challenge will next be to understand the differential diagenetic and catagenic losses and/or transformations that impact hopanoids and other hydrocarbons. Armed with such knowledge, geobiologists can begin to assess the patterns of ancient hydrocarbon deposition with greater confidence. Looking forward, we anticipate improvements in sampling and quantitative analysis will spur progress in assessing biomarker ratios from both modern and ancient ecosystems, facilitating a deeper understanding of the coevolution of life and Earth.

## Supporting Information

**Table S1.** Plasmids used in this study. **Table S2.** Primers used in this study. **Table S3.** Growth conditions for optimization of 2Me-BHT production in *R. palustris* TIE-1 WT and strain DKN1283. **Fig. S1.** Growth curves of *R. palustris* TIE-1 WT and strain DKN1283 in 96-well plates in the absence or presence of light in different growth media. **Fig. S2.** Optimization of 2Me-BHT production under different growth conditions evaluated by percentage of 2Me-BHT in total hopanoids (A and C), and the ratio of 2Me-BHT/BHT (B and D). Time course of these two factors from the cells grown in 50 mm MOPS, 20 mm succinate and 40 mm acetate is shown (E). Error bars represent standard deviation of three biological replicates. **Fig. S3.** LC-MS of purified hopanoids. **Fig. S4.** ^1^H NMR spectra of crude products in the conversions of **1** to **7** under the conditions indicated. **Data S1.** Characterization and experimental procedures.
