# Supplementary material for: Quantitative hopanoid analysis enables robust pattern detection and comparison between laboratories
Source: Geobiology. 2015 Apr 11;13(4):391–407. doi: 10.1111/gbi.12132 (PMC4676935; doi:10.1111/gbi.12132)
Supplement: Supplementary file 1 [file gbi0013-0391-sd1.pdf]

Supplementary Material

Quantitative hopanoid analysis enables robust pattern detection and comparison between laboratories

Content

	Page
**Supporting tables and figures**	S1
**General experimental information in chemical synthesis**	S8
**Construction of *hpnP* overexpression strain DKN1283**	S8
**Improvements on hopanoid purification from *R. palustris* TIE-1**	S9
**Large scale total lipid extraction and Hopanoid purification by silica gel and HPLC**	S9
**UPLC-ESI-TOF-MS analysis of hopanoids**	S10
**Screening of conditions for better production of 7**	S11
**Characterizations and experimental procedures**	S13
**References**	S25
**Appendix**	

Supporting tables and figures

Table S1: Plasmids used in this study

Plasmid	Description &/or Construction	Reference
pSRKGm	Complementation plasmid modified from pBBR1MCS-5; Gm^R	(Khan et al., 2008)
pJQ200KS	Mobilizable suicide vector; *sacB*, Gm^R	(Quandt and Hynes, 1993)
pAB314	pJQ200KS-derivative utilized for chromosomal integration of DNA at the *glmUSX-recG* locus of *R. palustris* TIE-1	(Bose and Newman, 2011)
pGK224	Cloned SphI/SmaI fragment of pAB314 without intergenic SpeI site into SphI/NotI-cut pJQ200KS using primers pAB314upfor, pAB314uprevfusion, pAB314dnforfusion, and pAB314dnrev	This study
pGK225	Cloned P*lac-lacI*-P*lac*-l*acZalpha* from pSRKGm flanked by Fd and T7 terminators into NcoI-cut pGK224 using primers lacIforw/Fd and lacIrevw/T7	This study
pGK226	Cloned *R. palustris* TIE-1 *hpnP* coding region into NdeI/AscI-cut pGK225 using hpnPfor and hpnPrev	This study

Table S2: Primers used in this study

Primer	Sequence	In bold
pAB314upfor	**GGCGCGCCGCATGC**CACACCGGCAGGTTG	AscI/SphI
pAB314uprevfusion	TTAGGTGCGGGTTAGTTACCACGCGTCATTTCGCGACCATGGCT ACCCGACCTTGTCCGG	-
pAB314dnforfusion	CCGGACAAGGTCGGGTAGCCATGGTCGCGAAATGACGCGTGGT AACTAACCCGCACCTAA	-
pAB314dnrev	**ACTAGTGCGGCCGC**CGAGATCGATTTTCTGGTCGGCAC	SpeI/NotI
lacIforw/Fd	**ACTAGTCCATGGTAAACCGATACAATTAAAGGCTCCTTTTGGAG CCTTTTTTTTTGGAG**TGATTGACACCATCGAATGGTG	SpeI/NcoI/Fd terminator
lacIrevw/T7	**ACTAGTCCATGGCAAAAAACCCCTCAAGACCCGTTTAGAGGCCC CAAGGGGTTATGGCGCGCC**TTACAATTTCCATTCGCCATTC	SpeI/NcoI/T7 terminator/AscI
hpnPfor	GAGATATA**CATATG**AAAGCCGAATCCGGGCAGA	NdeI
hpnPrev	**ACTAGTGGCGCGCC**CTACTCCGCCGGCACCGCCGCTTC	SpeI/AscI

Table S3. Growth conditions for optimization of 2Me-BHT production in *R. palustris* TIE-1 WT and strain DKN1283. Total lipid extract from each culture was analysed by GC-MS.

		% of all hopanoids				
**WT+light**	**OD_{600}**	**2Me-diplopterol (5)**	**Diplopterol (2)**	**2Me-BHT (15)**	**BHT (13)**	**%2MeBHT*OD_{600}**
YPMS	0.98	22	39	3	36	2.5
YPMS-B12	0.97	21	39	2	37	1.8
YPMS-Met	1.15	22	38	1	39	1.5
YPMS-squalene	1.01	18	34	1	47	1.4
YPMS-acetate	1.48	21	20	6	53	9.2
**WT w/o light**						
YPMS	0.40	16	49	2	33	0.7
YPMS-B12	0.45	18	43	3	35	1.4
YPMS-Met	0.41	12	46	2	40	0.7
YPMS-squalene	0.43	10	46	1	42	0.6
YPMS-acetate	0.63	19	32	4	46	2.3
**1283+light**						
YPMS	0.98	14	46	2	38	1.5
YPMS-B12	1.00	19	48	2	32	1.6
YPMS-Met	0.99	26	38	1	34	1.2
YPMS-squalene	1.08	20	48	2	31	1.9
YPMS-acetate	1.46	28	44	10	19	14.7
**1283 w/o light**						
YPMS	0.32	22	36	3	38	1.1
YPMS-B12	0.38	25	33	6	36	2.3
YPMS-Met	0.40	24	31	4	40	1.7
YPMS-squalene	0.40	22	38	3	37	1.1
YPMS-acetate	0.73	35	18	9	37	6.9

Figure S1. Growth curves of *R. palustris* TIE-1 WT and strain DKN1283 in 96-well plates in the absence or presence of light in different growth media. Error bars represent standard deviation of four replicates.

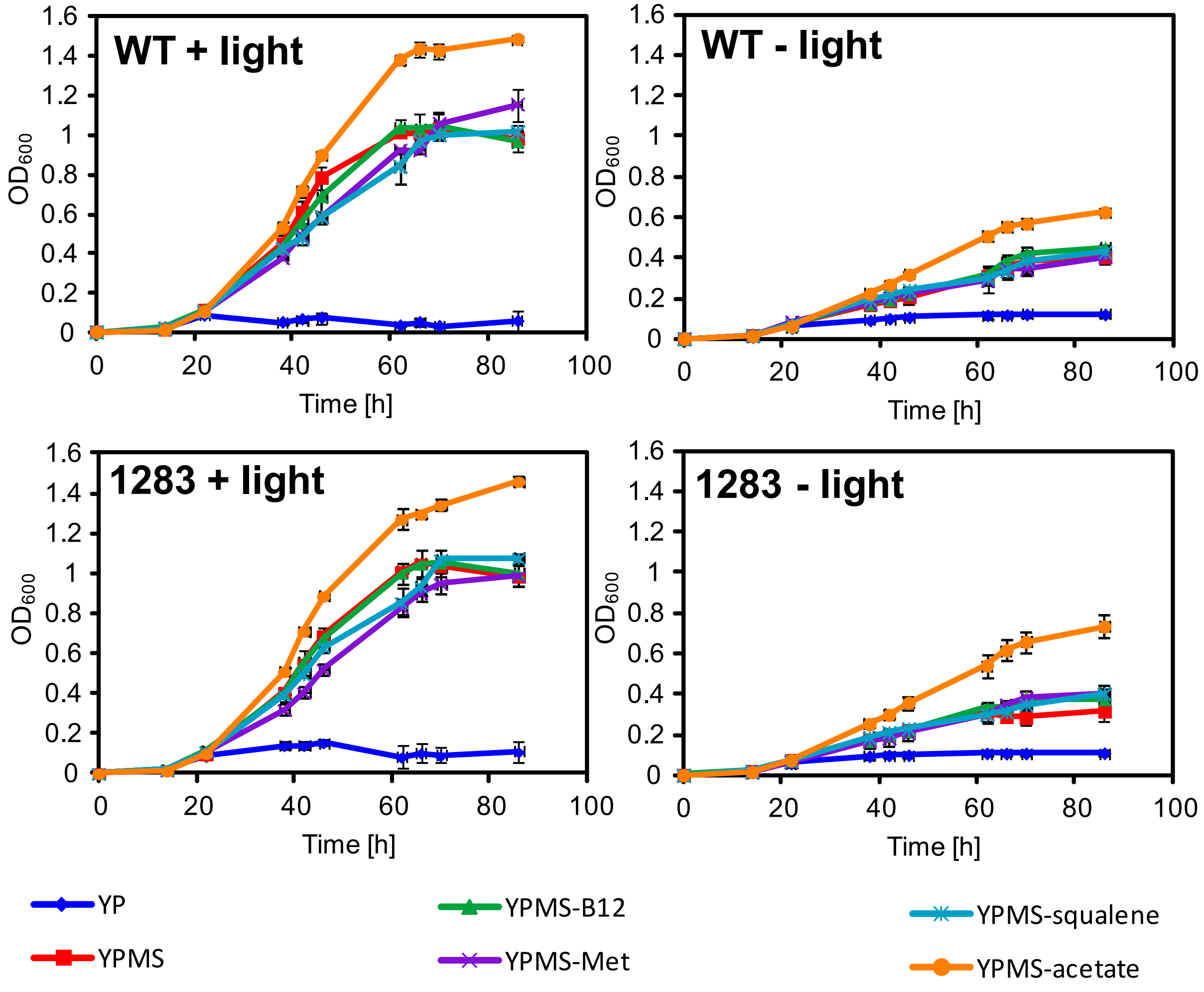

Figure S2. Optimization of 2Me-BHT production under different growth conditions evaluated by percentage of 2Me-BHT in total hopanoids (A and C), and the ratio of 2Me-BHT/BHT (B and D). Time course of these two factors from the cells grown in 50 mM MOPS, 20 mM succinate and 40 mM acetate is shown (E). Error bars represent standard deviation of three biological replicates.

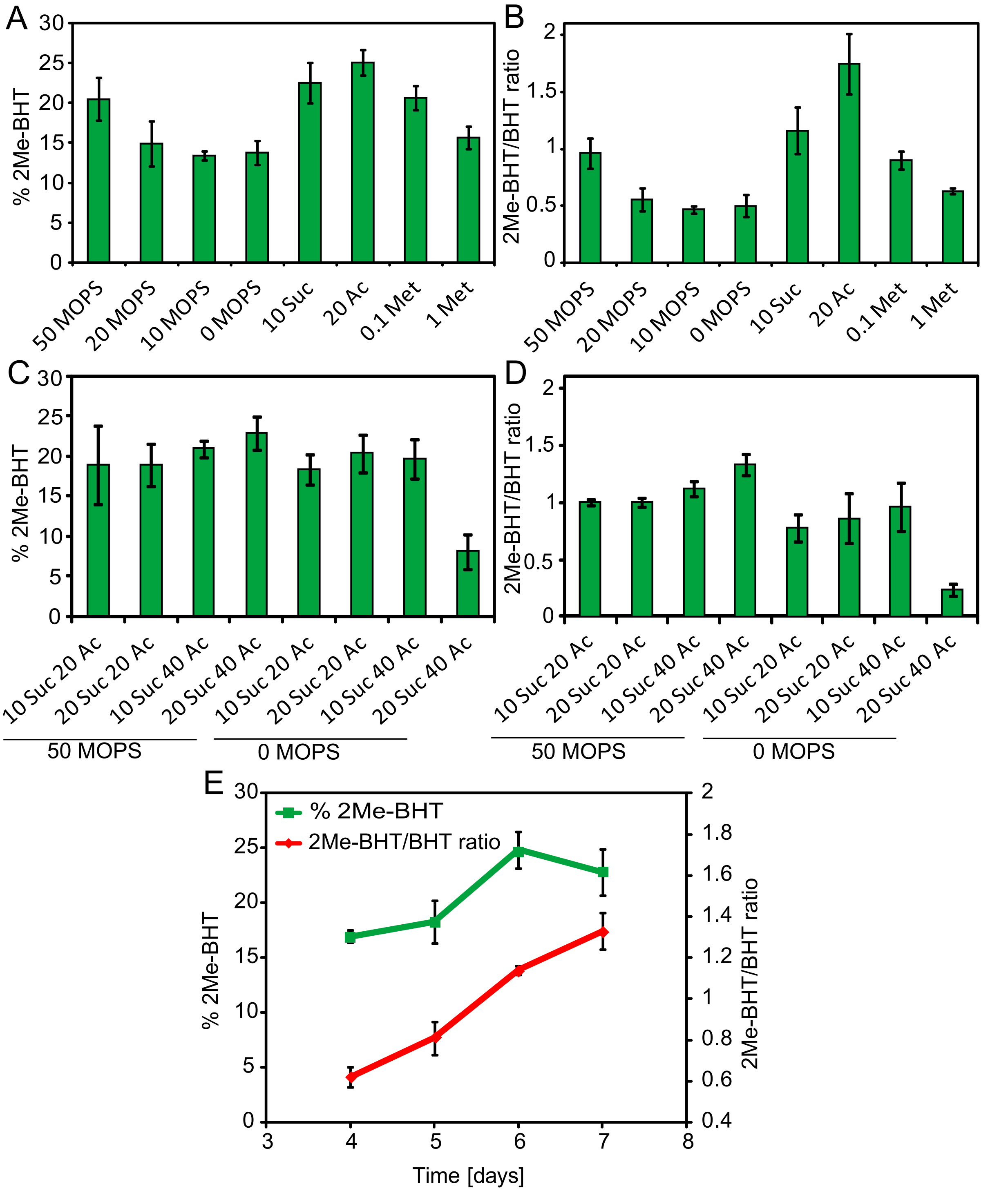

Figure S3: LC-MS of purified hopanoids. A/C: chromatograms of underivatized/acetylated samples; Y-axis shows the absolute signal intensity from MS; retention time and identity of the dominant peak is indicated in each chromatogram. B/D: MS spectra of the dominant peaks in A/C.

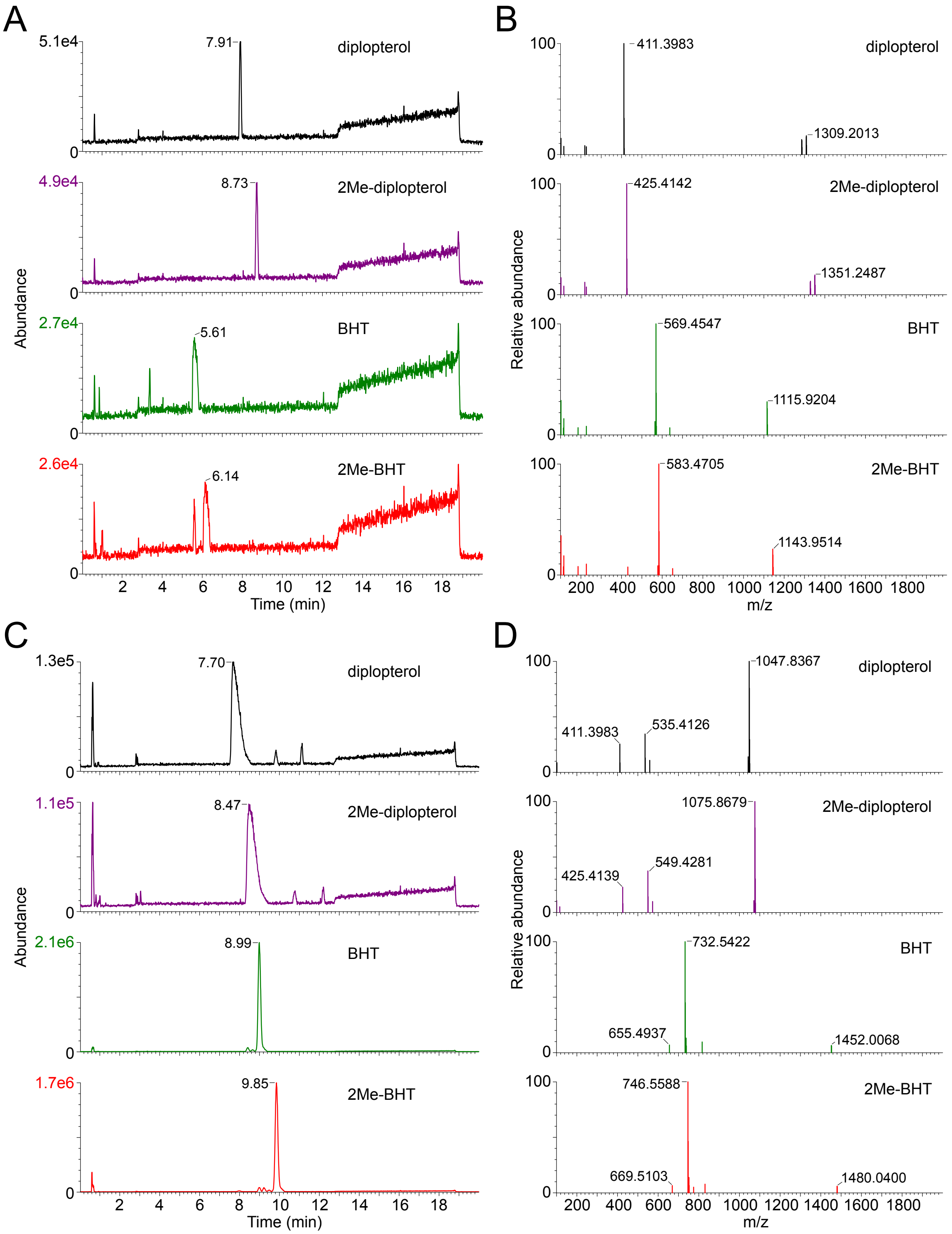

Figure S4. ^{1}H NMR spectra of crude products in the conversions of **1** to **7** under the conditions indicated.

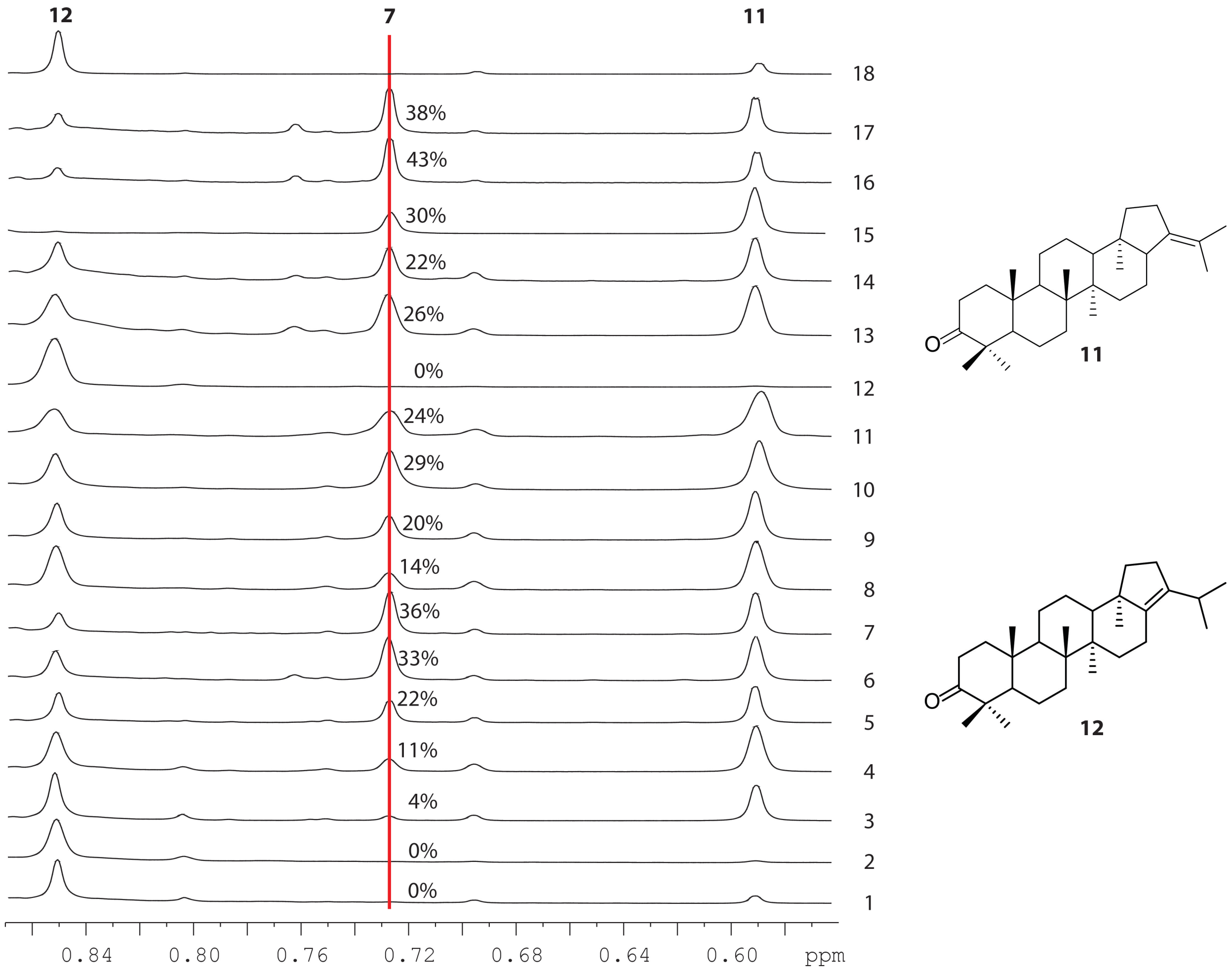

Note: For general and individual conditions, refer to “**Small-scale screening of conditions”.** The Lewis acid/catalysts used and temperatures applied are as follows: 1, $AlCl_3$, 0.25 eq., 65 °C; 2, $AlCl_3$ + $POCl_3$, 0.25 eq. of each reagent, 65 °C; 3, $InCl_3$, 0.25 eq., 65 °C; 4, $ZnCl_2$, 0.25 eq., 65 °C; 5, $ZrCl_4$, 0.25 eq., 65 °C; 6, CuCl, 0.25 eq., 65 °C; 7, $PdCl_2$, 0.25 eq., 65 °C; 8, $CoCl_2$, 65 °C; 9, CuCl, 0.5 eq., 65 °C; 10, $CuCl_2$, 0.25 eq., 65 °C; 11, $NiCl_2$, 0.25 eq., 65 °C; 12, $TiCl_4$, 0.25 eq., 65 °C; 13, $PdCl_2$, 0.25 eq., reflux; 14, $PdCl_2$, 1.25 eq. reflux; 15, $POCl_3$, excess (Dunstan et al., 1957); 16, $PdCl_2$, 0.25 eq., 80 °C; 17, $PdCl_2$, 1.25 eq. 80 °C; 18, Re_2O_7, 0.05 eq., RT(Korstanje et al., 2012).

General experimental information in chemical synthesis

Melting points were recorded on a Kofler hot stage and are uncorrected. Proton nuclear magnetic resonance spectra were recorded on a DRX 500 (500 MHz), Bruker AC 500 (500 MHz) or Bruker AV700 (700 MHz) spectrometer, as indicated. Carbon nuclear magnetic resonance spectra were recorded on a DRX 500 (126 MHz), Bruker AC 500 (126 MHz) or Bruker AV700 (176 MHz) with a 13C cryoprobe (125 MHz). Spectra were fully assigned using a combination of COSY, HSQC, and DEPT 135, HMBC. All chemical shifts were quoted on the scale in ppm using residual solvent as the internal standard. Coupling constants (*J*) are reported in Hertz (Hz). Identical proton coupling constants are averaged in each spectrum and reported to the nearest 0.1 Hz. Where appropriate, resonances for axial protons are denoted "*a*" and resonances for equatorial protons are denoted "*e*". Infrared spectra were recorded on a Bruker Tensor 27 Fourier Transform spectrophotometer with absorption maxima recorded in wavenumbers (cm^{-1}). Oils were analyzed as thin films. Low resolution mass spectra were recorded on an LCT Premier XE using electrospray ionization (ES). High resolution mass spectra were recorded on a Bruker micrOTOF. Specific rotations were measured on a Perkin Elmer 241 polarimeter with a pathlength of 1.0 dm with concentrations (c) given in g/100 mL (equivalent to g/0.1 dm^3). Specific rotations are denoted as $[\alpha]_D^T$ and are given in implied units of 10^{-1} deg cm^2 g^{-1} (T = ambient temperature in °C). Thin layer chromatography (TLC) was carried out on Merk Kieselgel 60F254 precoated aluminum backed plates and visualized with a combination of the following: 254 nm UV lamp; molybdate staining (10 g ammonium molybdate, 360 mL water, 40 mL 18N H_2SO_4). Flash column chromatography was carried out with Fluka Kiegselgel 60 220-440 mesh silica gel. Anhydrous solvents (Toluene, CH_2Cl_2) were dried according to the procedure outlined by Pangborn et al. (Pangborn et al., 1996) and used without purification unless otherwise indicated. All other solvents were used as supplied (analytical or HPLC grade), without further purification. Petroleum ether refers to the fraction of petroleum ether boiling in the range 40-60 ºC. Reagents and solvents used, unless otherwise stated, were of commercially available reagent grade quality and were used without further purification.

Construction of *hpnP* overexpression strain DKN1283

In an attempt to increase the production of 2-methylhopanoids, we expressed *hpnP* from an IPTG-inducible promoter on the chromosome. A modified version of the pAB314-based homologous recombination system was used to integrate *lac* promoter (P*lac*) driven *hpnP* at the permissive intergenic region of Rpal_2346 (*glmX*) and Rpal_2396 (*recG*). For this, the 2 Kb region around *glmX*-*recG* with a mutated SpeI site was sub-cloned from pAB314 into the parent plasmid pJQ200KS to give pGK224. The

lac repressor along with the IPTG-inducible P*lac* (P*lac*-*lacI*-P*lac*-l*acZalpha*) with flanking transcriptional terminators Fd and T7, was then sub-cloned from pSRKGm into pGK224 to give pGK225. Finally, the *hpnP* coding region was cloned downstream of P*lac* to give the overexpression plasmid pGK226. pGK226 was transformed into WT *R. palustris* TIE-1 using gentamicin selection. The pGK226-integrants were then resolved using sucrose counterselection to obtain markerless DKN1283.

Improvements on hopanoid purification from *R. palustris* TIE-1

To purify hopanoids, we first applied protocols for analytical scale purification (Welander et al., 2012), which use solvents with increasing polarity to elute, in sequence, diploptene (**8**), (2Me)-diplopterol (**2** and **5**), and (2Me)-BHT (**13** and **15**). However, the dissolution of silica gel became a problem when using 4:1 EtOAc:MeOH. Unfortunately, this silica gel contamination could not be separated completely and replacing methanol with other polar solvents like acetone caused the same problem. To circumvent this issue, we derivatized the hydroxyl groups in (2Me)-BHT with a UV-absorptive moiety such as benzoyl chloride, phenyl acetyl chloride, or hydrocinnamoyl chloride, to decrease BHT's polarity and permit detection by HPLC equipped with a UV-vis detector. However, the heterogeneity of the products prohibited a clean separation between methylated and unmethylated hopanoids. One primary alcohol-specific derivatization by tert-butyl(chloro)diphenylsilane was tested but the addition of a large functional group compromised the separation between 2Me-BHT and BHT. We then tested acetylation of hopanoids, which not only changed their polarity but also increased the solubility of (2Me)-BHT in DCM (CH_2Cl_2) dramatically, allowing more sample to be loaded onto the HPLC column. However, the acetylation of total lipid extract produced diplopterols with multiple acetyl groups that made the subsequent recovery of diplopterols after de-acetylation difficult. Therefore, our final protocol was to purify diplopterols from un-derivatized TLE and to acetylate the rest of the sample to purify acetylated BHTs. Silica gel purified (2Me)-diplopterol and acetylated (2Me)-BHT were then purified by HPLC using a single solvent composition. The final purified (2Me)-BHT was obtained after de-acetylation.

Large scale total lipid extraction and hopanoid purification by silica gel and HPLC

Briefly, the optimized conditions for extraction and purification of various hopanoids are as follows. Total lipid extract (TLE) from *R. palustris* TIE-1 strain DKN1283 was prepared using the Bligh-Dyer method (Bligh and Dyer, 1959) and modified for large-scale extraction. In a 4 L glass beaker, cell paste was suspended in water to reach a final volume of 5 mL solution per gram of wet cell paste. Then the equivalent of 12.5 mL methanol (MeOH) and 6.25 mL CH_2Cl_2 per gram of wet cell paste were added in sequence and stirred for 1 h before sonication for a total pulse time of 1 h while stirring (1/8 inch tip, power output 3.5, 1 sec on, 4 sec off, 4 °C, Sonic

Dismembrator 550, Fisher Scientific). After the sonicated sample was warmed to room temperature (RT), CH_2Cl_2 was added at a volume ratio of sample: CH_2Cl_2=4:3. After gentle stirring, the sample was allowed to settle for a few hours to overnight, permitting the organic and aqueous layer to separate. The bottom organic layer (dark brown) was collected and dried in a rotary evaporator. A typical dark green TLE was obtained. For silica gel purification, TLE was dissolved in CH_2Cl_2 and adsorbed on to Celite 545 (4 g per 1 g of TLE) using a rotary evaporator. About 7 g of Celite-adsorbed TLE was loaded onto a column packed with ~300 mL of silica gel (~25 cm × 4 cm) pre-equilibrated with hexane (Hex). The column was eluted in sequence of ~450 mL hexane, ~600 mL 3:1 hexanes:EtOAc, and ~200 mL pyridine. The presence of diploptene, diplopterols, and BHTs in each respective elution was monitored by TLC plates and visualized by molybdate staining (10 g ammonium molybdate, 360 mL water, 40 mL 9M H_2SO_4) after heating on a hot plate (diploptene Rf~0.97, (2Me)-diplopterol (**2** and **5**) Rf~0.51 in 5:1 hexanes:EtOAc, and (2Me)-BHT Rf~0.88 in 1:8:10 water:IPA:EtOAc). As the solubility of (2Me)-BHT (**13** and **15**) is too low for downstream HPLC purification, the sample was acetylated to increase solubility. The fractions containing (2Me)-BHT were dried in a rotary evaporator and acetylated in 20 mL pyridine and 20 mL acetic anhydride at 60 °C for ~1.5 h. The acetylated BHTs were dried in the presence of Celite and packed onto a silica gel column as previously described. The column was equilibrated with ~500 mL of 8:1 hexanes:EtOAc and eluted with ~500 mL of 5:1 hexanes:EtOAc and then ~500 mL of 4:1 hexanes:EtOAc. The presence of (2Me)-BHT-4Ac was monitored by the same TLC method as above (Rf~0.21 in 5:1 hexanes:EtOAc). Further separation between methylated and unmethylated hopanoids was carried out using reverse phase HPLC (Phenomenex Luna C18(2), 100 Å, 5 μm, 250 × 21.2 mm column coupled with a Shimadzu LC-8A preparatory pump and a Beckman SC 100 fraction collector). The silica gel purified (2Me)-diplopterol samples were dried and re-dissolved at 1 g per mL of CH_2Cl_2 and about 100 mg was injected into the HPLC, pre-equilibrated in 95:5 MeOH:water and eluted with the same solvent at 20 mL/min. The silica gel purified (2Me)-BHT-4Ac were purified by HPLC as above except the column was pre-equilibrated in 65:35 IPA:water and eluted with the same solvent at 12 mL/min. HPLC-purified BHT-4Ac (**14**) and 2Me-BHT-4Ac were collected and dried in a rotary evaporator. The samples were de-acetylated by dissolution in 1:1 CH_2Cl_2:MeOH with a substoichiometric amount of sodium methoxide and incubated stirring at RT overnight. The products were passed through an Amberlite IR-120 column (hydrogen form, washed extensively with water, then dried with acetone, and packed in MeOH) and eluted with MeOH to exchange the by-product sodium acetate to acetic acid, which was subsequently removed along with solvent in a rotary evaporator.

UPLC-ESI-TOF-MS analysis of hopanoids

To analyze the unacetylated or acetylated hopanoids by LC-MS, protocols based on Waters Application Note with modifications were followed (Isaac et al., 2011). The samples were dissolved sonication in isopropanol:acetonitrile:water (2:1:1) at a final concentration of 1 mg/mL. The samples (5 μl/injection, three replicates per sample, all in randomized order) were separated by a charged surface hybrid C_{18} column (Waters Acquity UPLC CSH C_{18}, 2.1 x 100 mm, 1.7 μm) and analyzed by Waters LC-MS/MS system (Acquity I-Class UPLC with Xevo G2-S TOF). The column temperature was maintained at 55 °C. A binary solvent system containing solvent A (acetonitrile:water; 60:40) and solvent B (isopropanol:acetonitrile; 90:10), both with 10 mM ammonium formate and 0.1% formic acid was used. The flow rate was set at 400 μL/min and the elution program started at 40% B, increased linearly to 43% B in 2 min, then to 50% B in 0.1 min, followed by linear increase to 54% B over 9.9 min, then jumped to 70% B in 0.1 min, increased linearly to 99% B over 5.9 min, then decreased immediately to 40% B in 0.1 min, and maintained at the same level for 1.9 min.

The eluents from the column were ionized by electrospray ionization (ESI). MS^E data from 100 to 1500 m/z was collected in either positive or negative ion mode. MS^E consists of both low energy and high energy scans obtained simultaneously. During data analysis product ions can be associated with parent ions if they are coincident in chromatographic time. Electrospray conditions were capillary voltage 2.0 kV, cone voltage 30 V, source offset 60 V, source temperature 120 °C, desolvation temperature 550 °C, cone gas 20 L/h, and desolvation gas 900 L/h. The TOF-MS was operated in resolution mode, typically 32,000 m/Δm. The mass axis was calibrated with sodium formate clusters. Leucine enkephalin was used as a mass reference during acquisition. The data were collected in continuum mode, and then converted to centroid mode for quantitative analysis using the Quanlynx program (Waters Corporation, Milford, MA).

Screening of conditions for better production of 3-Oxo-diploptene (7)

Small-scale screening of conditions:

Conditions 1-17 in Figure S4: **1** (5 mg, 12 μmol) was dissolved in anhydrous toluene (2 mL) with 3 Å molecular sieves (MS). The Lewis acid/catalyst was added to the solution which was then heated to 65/80 °C, or under reflux. TLC analysis was employed to monitor the reactions. Typically, full conversion of **1** was observed within 2 hours. The MS and catalysts were filtered through Celite, followed by washing with CH_2Cl_2 and concentration *in vacuo*. The resulting solid was dried *in vacuo* and then dissolved in CD_2Cl_2 and subjected to ^{1}H NMR analysis.

Condition 18 in Figure S4: **1** (30 mg, 68 μmol) was dissolved in anhydrous toluene (5 mL). Re_2O_7 (Corbett and Smith, 1967) (1.7 mg, 3.5 μmol) was added to the solution, which was then heated to 100 °C. Compound **1** was fully consumed, as indicated by

TLC analysis. The solution was filtered through Celite, followed by washing with CH_2Cl_2 and concentration *in vacuo*. The resulting solid was dried *in vacuo* and then dissolved in CD_2Cl_2 and subjected to ^{1}H NMR analysis.

The optimum condition in small-scale screening was found to be 0.25 eq. $PdCl_2$, 80 °C, 2 h, which gave 43% of **7** in the crude products.

Large-scale optimization

After further trials on larger scales, it was found that the optimum conditions for converting 1-2 gram of **1** to **7** were 0.5 eq. $PdCl_2$, 70 °C, 5 h, which gave ~40% (yield obtained after Ag coated silica chromatography followed by crystallization) of **7** from CH_2Cl_2 and hexane.

Characterization and experimental procedures

Hydroxyhopanone-C30 (1)

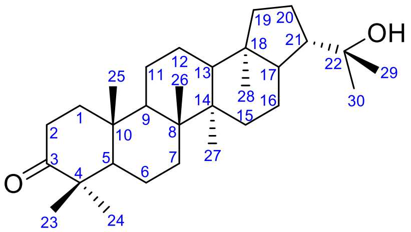

Dammar resin (1 kg) was ground into powder, which was poured into methanol (6 L) (Dunstan et al., 1957). The suspension was boiled for 1 hour with stirring and then left overnight without stirring at RT. Undissolved materials were removed by filtration. The solvent was evaporated on a rotary evaporator. The resulting solid was dissolved in diethyl ether (8 L). The organic solution was washed with an aqueous solution of potassium hydroxide (5%, 1.5 L) and then water. The resulting organic solution was dried over magnesium sulfate, filtered and the filtrate was transferred into 4 glass bottles (2 L). The bottles were then placed on ice. Half of the solvent was removed under a slow, continuous stream of nitrogen. The remaining solution was combined into a 4 L bottle. The solvent was further evaporated on ice until ~1 L solution was left. Colorless solids were found attached on the inner walls of the bottle. The bottle was sealed and placed in a fridge. After three days, the precipitate was removed by filtration. The resultant colorless solid was washed with a small amount of cold ether. The solid was dissolved in ether and slowly crystallized in methanol. The crystals were then collected and dried to afford hydroxyhopanone (**1**) as colorless needles (7.7 g, 0.77 %): R_f 0.35 (hexanes: diethyl ether = 1: 1); $[\alpha]^{20}_{D}$ = +70 (*c* = 0.5, dichloromethane) (Lit. +64 (Dunstan et al., 1957)); m.p. 275-277 °C (diethyl ether) (Lit. 252-256 °C, (acetone) (Dunstan et al., 1957)); ν_{max} (ATR)/cm^{-1} 3469, 2991 ($\nu_{O\text{-}H}$), 2946, 2888, 2863 ($\nu_{C\text{-}H}$), 1708 ($\nu_{C=O}$), 1444, 1377 ($\nu_{C\text{-}H}$), 1147 ($\nu_{C\text{-}O}$), 828 ($\nu_{C\text{-}H}$); ^{1}H NMR (700 MHz, CD_2Cl_2) δ 0.76 (s, 3H, H-28), 0.93 (s, 3H, H-25), 0.95 (d, 1H, *J* 2.5 Hz, H-19*a*), 0.96 (s, 3H, H-27), 0.99 (s, 3H, H-23), 1.01 (s, 3H, H-26), 1.04 (s, 3H, H-24), 1.14 (s, 3H, H-30), 1.18 (s, 3H, H-29), 1.24-1.29 (m, 2H, H-15*a* & H-7*e*), 1.29-1.35 (m, 2H, H-5 & H-9), 1.36-1.55 (m, 13H, H-11*e*, H-1*e*, H-13, H-15*e*, H-12*a*, H-6*a*, H-12*e*, H-17, H-20*a*, H-6*e*, H-11*a*, H-7*a*, H-19*e*), 1.71-1.78 (m, 1H, H-20*e*), 1.89-1.96 (m, 2H, H-16*e* & H-1*a*), 2.20 (dt, 1H, *J* 9.0, 11.0 Hz, H-21), 2.32-2.37 (m, 1H, , H-2*e*), 2.48 (m, 1H, H-2*a*); ^{13}C NMR (176 MHz, CD_2Cl_2) δ 15.9 (C-25), 16.4 (C-28), 16.6 (C-26), 17.0 (C-27), 20.1 (C-6), 21.3 (C-23), 21.9 (C-11), 22.3 (C-16), 24.5 (C-12), 26.7 (C-24), 26.9 (C-20), 29.0 (C-30), 31.1 (C-29), 33.0 (C-7), 34.6 (C-2), 34.8 (C-15), 37.2 (C-10), 39.9 (C-1), 41.6 (C-19), 42.0 (C-8), 42.3 (C-14), 44.4 (C-18), 47.6 (C-4), 50.0 (C-9), 50.4 (C-13), 51.5 (C-21), 54.4 (C-17), 55.2 (C-5), 73.9 (C-22), 217.8 (C-3); HRMS *m/z* (FI) [Calculated for $C_{30}H_{50}O_2$ 424.3811; found 424.3897].

Diplopterol (hydroxyhopane, 2)

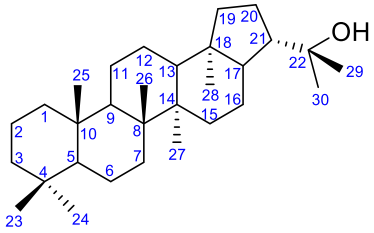 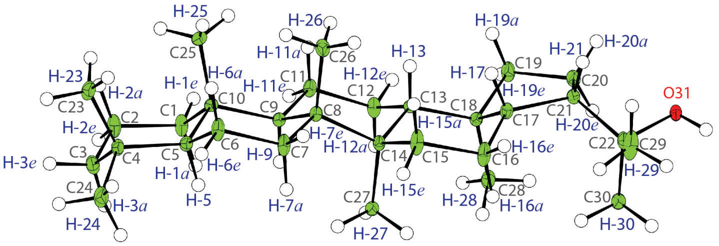

To **1** (500 mg, 1.13 mmol, 1.0 eq.) in a glass flask was added diethylene glycol (50 mL) and hydrazine hydrate (3.6 mL, 72 mmol, 62 eq.) under reflux for 2 hours (Dunstan et al., 1957). The excess of hydrazine and water was removed by distillation. Potassium hydroxide (1.3 g, 23 mmol, 20 eq.) was added and the solution was heated to 200-210 ºC for 6 hours. After dilution with water, the product was extracted by an ether/water extraction. Evaporation of diethyl ether afforded a solid that was crystallized from acetone-methanol to afford diplopterol **2** as colorless solid (462 mg, 95%): R_f 0.55 (hexanes: diethyl ether = 1: 1); $[\alpha]_D^{20}$ = +47.4 (*c* = 0.5, diethyl ether) (Lit. +45 (Dunstan et al., 1957)); m.p. 252-254 °C (diethyl ether-methanol) (Lit. 254-255 °C, acetone-methanol (Dunstan et al., 1957)); ν_{max} (ATR)/cm^{-1} 3385, 2991(ν_{O-H}), 2944, 2866 (ν_{C-H}), 1459, 1443, 1387, 1376 (ν_{C-H}), 1130 (ν_{C-O}); ^{1}H NMR (700 MHz, CD_2Cl_2) δ 0.74 (dd, 1H, *J* 1.5, 12.0 Hz, H-5), 0.76 (s, 3H, H-28), 0.76-0.81 (m, 1H, H-1*a*), 0.80 (s, 3H, H-23), 0.82 (s, 3H, H-25), 0.85 (s, 3H, H-24), 0.95 (dd, 1H, *J* 2.8, 12.4 Hz, H-19*a*) 0.96 (s, 3H, H-27), 0.97 (s, 3H, H-26), 1.10-1.16 (m, 1H, H-3*a*), 1.14 (s, 3H, H-30), 1.18 (s, 3H, H-29), 1.19-1.28 (m, 3H, H-7a, H-15*a*, H-9), 1.29-1.43 (m, 7H, H-12*a*, H-6*a*, H-3*e*, H-2*e*, H-13, H-15*e*, H-1*e*), 1.43-1.63 (m, 8 H, H-17, H-11*a*, H-7*a*, H-20*a*, H-19*e*, H-12*e*, H-16*a*, H-2*a*), 1.65 (dt, 1H, *J* 3.5, 12.7 Hz, H-1*e*), 1.70-1.77 (m, 1H, H-20*e*), 1.92 (m, H-16*e*), 2.20 (m, 1H, H-21); ^{13}C NMR (125 MHz, CD_2Cl_2) δ 16.0 (C-25), 16.3 (C-28), 16.9 (C-26), 17.2 (C-27), 19.10 (C-6), 19.11 (C-2), 21.3 (C-12), 21.7 (C-23), 22.3 (C-16), 24.5 (C-11), 27.0 (C-20), 29.0 (C-30), 31.1, (C-29), 33.54 (C-4), 35.55 (C-24), 33.61 (C-7), 34.8 (C-15), 37.8 (C-10), 40.7 (C-1), 41.6 (C-19), 42.2 (C-8), 42.3 (C-14), 42.5 (C-3), 44.5 (C-18), 50.2 (C-13), 50.8 (C-9), 51.6 (C-21), 54.3 (C-17), 56.4 (C-5), 74.0 (C-22). HRMS *m/z* (FI) [Calculated for $C_{30}H_{52}O$ 428.4018; found 428.4015].

2,2-D_2-Hydroxyhopanone (3)

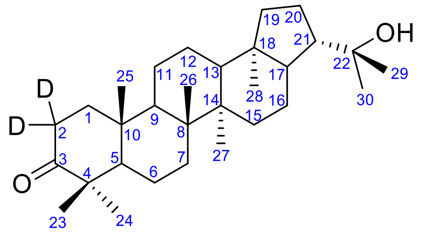

Compound **1** (250 mg, 0.57 mmol, 1.0 eq.) was dissolved in CD_2Cl_2 (15 mL), MeOD (5.0 mL) and deuterium oxide (D_2O, 0.75 mL) in a glass flask. NaOD in D_2O (40 wt.%, 40 µL, 1.0 eq.) was added. The reaction was kept overnight under stirring and then quenched by a dry carbon dioxide flow. The deuterated product was extracted by an ether/water extraction. The organic fraction was dried over magnesium sulfate and filtered. The solvent was removed from the filtrate on a rotary evaporator to afford product **3** as colorless crystals in 100% yield (250 mg): R_f 0.35 (hexanes: diethyl ether = 1: 1); $[\alpha]_D^{20}$ = +61.2 (*c* = 0.5, dichloromethane); m.p. 254-257 (CH_2Cl_2); ν_{max} (ATR)/cm^{-1} 3472 (ν_{O-H}), 2991, 2946, 2890, 2856 (ν_{C-H}), 1704 (ν_{C-O}), 1444, 1377 (ν_{C-H}), 1149,1081 (ν_{C-O}), 827 (ν_{C-H}); ^{1}H NMR (700 MHz, CD_2Cl_2) δ 0.76 (s, 3H, H-28), 0.93 (s, 3H, H-25), 0.95 (d, 1H, *J* 2.5 Hz, H-19*a*), 0.96 (s, 3H, H-27), 0.99 (s, 3H, H-23), 1.01 (s, 3H, H-26), 1.04 (s, 3H, H-24), 1.14 (s, 3H, H-30), 1.18 (s, 3H, H-29), 1.24-1.29 (m, 2H, H-15*a* & H-7*e*), 1.29-1.35 (m, 2H, H-5 & H-9), 1.36-1.55 (m, 13H, H-11*e*, H-1*e*, H-13, H-15*e*, H-12*a*, H-6*a*, H-12*e*, H-17, H-20*a*, H-6*e*, H-11*a*, H-7*a*, H-19*e*), 1.69-1.79 (m, 1H, H-20*e*), 1.91 (d, 1H, *J* 13.2 Hz, H-1*a*), 1.87-1.97 (m, 1H, H-16*e*), 2.20 (dt, 1H, *J* 9.0, 11.1 Hz, H-21); ^{13}C NMR (125 MHz, CD_2Cl_2) δ 15.9 (C-25), 16.4 (C-28), 16.6 (C-26), 17.0 (C-27), 20.1 (C-6), 21.3 (C-23), 21.9 (C-11), 22.3 (C-16), 24.5 (C-12), 26.7 (C-24), 26.9 (C-20), 29.0 (C-30), 31.1 (C-29), 33.0 (C-7), 34.8 (C-15), 37.2 (C-10), 39.8 (C-1), 41.6 (C-19), 42.0 (C-8), 42.3 (C-14), 44.4 (C-18), 47.6 (C-4), 50.0 (C-9), 50.4 (C-13), 51.5 (C-21), 54.4 (C-17), 55.2 (C-5), 73.9 (C-22), 217.9 (C-3); HRMS *m/z* (FI) [Calculated for $C_{30}H_{48}D_2O_2$ 444.3936; found 428.3932]; Calculated with the signals in mass spectrometry using Force Ionization (FI) D2:D1:D0 = 86%: 18%: 6%.

2,2,3,3-D_4-Diplopterol (4)

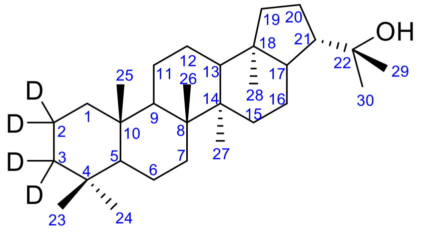

Compound **3** (145 mg, 326 µmol, 1.0 eq.) was dissolved in a mixture of MeOD and D_6-toluene (10 + 20 mL). 4-Toluenesulfonyl hydrazide (303 mg, 1.63 mmol, 5.0 eq.) was added to the solution, followed by heating under reflux with a Soxhlet condenser filled with activated 3 Å molecular sieves. After 4 hours, the solution was cooled to RT.

NaBD$_4$ (27 mg, 652 µmol, 2.0 eq.) was added. After heating under reflux for a further 2 hours, the solution was cooled to RT and concentrated on a rotary evaporator. The resulting solid was subjected to silica chromatography (petroleum ether: diethyl ether = 11: 2) to afford compound **4** as a colorless solid (62 mg, 44%): R_f 0.55 (hexanes: diethyl ether = 1: 1); $[\alpha]_D^{20}$ = +36.5 (*c* = 0.2, diethyl ether); m.p. 253-255 °C (diethyl ether); ν_{max} (ATR)/cm^{-1} 3472 (ν_{O-H}), 2946, 2867 (ν_{C-H}), 1466, 1386, 1372 (ν_{C-H}); ^{1}H NMR (500 MHz, CD_2Cl_2) δ 0.73 (dd, 1H, *J* 1.8, 12.3 Hz, H-5), 0.75 (s, 3H, H-28), 0.74-0.78 (m, 1H, H-1*a*), 0.79 (s, 3H, H-23), 0.82 (s, 3H, H-25), 0.84 (s, 3H, H-24), 0.90-0.95 (m, 1H, H-19*a*) 0.96 (s, 3H, H-27), 0.97 (s, 3H, H-26), 1.14 (s, 3H, H-30), 1.17 (s, 3H, H-29), 1.18-1.28 (m, 3H, H-7*e*, H-15*a*, H-9), 1.29-1.44 (m, 5H, H-12*a*, H-6*a*, H-13, H-15*e*, H-11*e*), 1.44-1.56 (m, 5 H, H-17, H-11*a*, H-7*a*, H-20*a*, H-12*e*), 1.58 (dd, *J* 3.6, 12.3 Hz, H-16*a*), 1.60-1.67 (m, 1H, H-1*e*), 1.69-1.78 (m, 1H, H-20*e*), 1.88-1.95 (m, H-16*e*), 2.15-2.25 (m, 1H, H-21); ^{13}C NMR (125 MHz, CD_2Cl_2) δ 16.2 (C-25), 16.5 (C-28), 17.1 (C-26), 17.3 (C-27), 19.2 (C-6), 21.5 (C-12), 21.9 (C-23), 22.5 (C-16), 24.7 (C-11), 27.1 (C-20), 29.1 (C-30), 31.3 (C-29), 33.5 (C-4), 33.6 (C-24), 33.8 (C-7), 34.9 (C-15), 37.9 (C-10), 40.6 (C-1*), 40.7 (C-1), 41.8 (C-19), 42.38, 42.43 (C-8, C-14), 44.6 (C-18), 50.4 (C-13), 50.9 (C-9), 51.7 (C-21), 54.5 (C-17), 56.5 (C-5), 74.1 (C-22); HRMS *m/z* (FI) [Calculated for $C30H_{48}D_4O$ 432.4269; found 432.4484].

C1 of the tri-deuterated compound (C1:C1= ~3:1). Calculated with the signals in mass spectrometry using Force Ionization (FI) D4:D3:D2 = 55%: 37%: 8%.

(3*S*)-3-Hydroxydiplopterol (6)

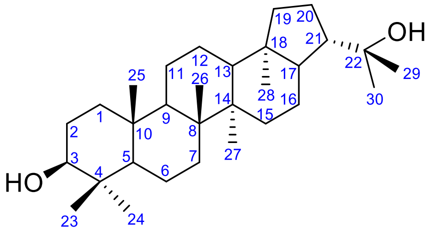

Compound **1** (100 mg, 226 µmol, 1.0 eq.) was dissolved in methanol and diethyl ether (10 mL;10 mL). NaBH$_4$ (85 mg, 2.2 mmol, 9.7 eq.) was added. The reaction was stirred at RT for 1 h. The solvent was evaporated. Diethyl ether (50 mL) and water (50 mL) were added to the resulting solid. The product was extracted with further diethyl ether (2 × 50 mL). The organic phase was dried over anhydrous MaSO$_4$, and filtered. The solvent was removed on a rotary evaporator, followed by crystallization from diethyl ether, to give **6** as small colorless needles (90 mg, 90%): R_f 0.22 (hexanes: diethyl ether = 1: 1); $[\alpha]_D^{20}$ = +38 (*c* = 0.15, CH_2Cl_2); m.p. 256-258 °C (diethyl ether); ν_{max} (ATR)/cm^{-1} 3366 (ν_{N-H}, ν_{O-H}), 2942 (ν_{C-H}), 1448, 1374 (ν_{C-H}); ^{1}H NMR (500 MHz, CD_2Cl_2) δ 0.69 (dd, 1H, *J* 1.8, 11.9 Hz, H-5), 0.74 (s, 3H, C-24), 0.75 (s, 3H, C-28), 0.82 (s, 3H, C-25), 0.92-0.98 (m, 11H, H-1*a*, H-19*a*, H-26, H-27, H-23), 1.14 (s, 3H, H-30), 1.17 (s, 3H, H-29), 1.20-1.31 (m, 3H, H-9, H-7*e*, H-15*a*), 1.31-1.63 (m, 15H, H-12*a*, H-6*a*, H-11*e*, H-13, H-15*e*, H-17, H-20*a*, H-7*a*, H-11*a*, H-6*e*, H-12*e*, H-19*e*, H-2, H-16*a*), 1.65-1.78 (m, 2H, H-1*e*, H-20*e*), 1.89-1.96 (m, 1H, H-16*e*), 2.15-2.24 (m, 1H, H-21), 3.11-3.19 (m, 1H, H-3);

^{13}C NMR (125 MHz, CD_2Cl_2) δ 15.6 (C-23), 16.1 (C-25), 16.4 (C-28), 16.9 (C-26), 17.2 (C-27), 18.9 (C-6), 21.5 (C-12), 22.4 (C-16), 24.5 (C-11), 27.0 (C-20), 28.0 (C-2), 28.3 (C-23), 29.1 (C-30), 31.2 (C-29), 33.7 (C-7), 34.9 (C-15), 37.6 (C-10), 39.1 (C-1), 39.2 (C-4), 41.7 (C-19), 42.2 (C-8), 42.3 (C-14), 44.5 (C-18), 50.4 (C-13), 50.8 (C-9), 51.6 (C-21), 54.4 (C-17), 55.5 (C-5), 74.0 (C-22), 79.2 (C-3); HRMS *m/z* (FI) [Calculated for $C_{30}H_{52}O_2$ 444.3967; found 444.4068].

(2*S*)-2-Methyldiplopterol (5)

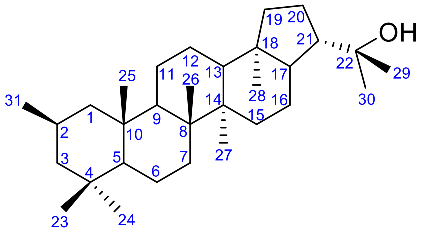

Refer to **Large scale total lipid extraction and Hopanoid purification by silica gel and HPLC** (Page S9) for purification procedure. Characterization of **5**: R_f 0.55 (hexanes: diethyl ether = 1: 1); $[\alpha]_D^{20}$ = +88.8 (*c* = 0.17, diethyl ether); m.p. 193-195 °C (CH_2Cl_2)); ν_{max} (ATR)/cm^{-1} 3472 (ν_{O-H}), 2946, 2867 (ν_{C-H}), 1466, 1386, 1372 (ν_{C-H}); ^{1}H NMR (500 MHz, CD_2Cl_2) δ 0.76 (H-28), 0.82 (d, 3H, *J* 6.0 Hz, H-31), 0.83 (s, 3H, H-25), 0.85 (dd, 1H, *J* 2.8, 13.9 Hz, H-1*a*), 0.88 (s, 3H, H-24), 0.89 (s, 3H, H-23), 0.95 (br s, 6H, H-24, H-27), 0.96 (s, 3H, H-26), 0.91 (dd, 1H, *J* 4.1, 8.8 Hz, H-5), 0.96 (dd, 1H, *J* 6.4, 8.8 Hz, H-19*a*), 1.10-1.15 (m, 1H, H-3*e*), 1.14 (s, 3H, H-30), 1.17 (s, 3H, H-29), 1.18-1.30 (m, 4H, H-7*e*, H-3*a*, H-15*a*, H-9), 1.34-1.62 (m, 14H, H-12*a*, H-6*a*, H-13, H-15*e*, H-6*e*, H-11*e*, H-7*a*, H-17, H-1*e*, H-11*a*, H-12*e*, H-20*a*, H-19*e*, H-16*a*), 1.55-1.63 (m, 1H, H-2), 1.70-1.77 (m, 1H, H-20*e*), 1.89-1.95 (m, 1H, H-16*e*), 2.15-2.24 (m, 1H, H-21); ^{13}C NMR (125 MHz, CD_2Cl_2) δ 16.6 (C-26, C-28),16.3 (C-27), 20.5 (C-6), 22.1 (C-23), 22.4 (C-12), 22.5 (C-16), 23.5 (C-31), 25.0 (C-11), 25.3 (C-2), 26.4 (C-25), 27.1 (C-20), 29.1 (C-30), 31.27 (C-24), 31.29 (C-29), 32.9 (C-4), 33.0 (C-7), 35.0 (C-15), 38.3 (C-10), 41.8 (C-19), 42.4 (C-8), 42.5 (C-14), 44.6 (C-18), 45.7 (C-3), 50.1 (C-5), 50.3 (C-1), 50.9 (C-13), 51.4 (C-9), 51.7 (C-21), 54.5 (C-17), 74.1 (C-22); HRMS *m/z* (FI) [Calculated for $C_{31}H_{54}O$ 442.4175; found 442.4190].

3-Oxo-diploptene (7) and 3-oxo-21-ene-deoxy-hopane (11)

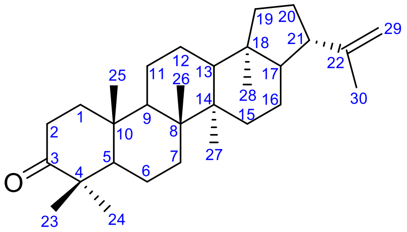

7

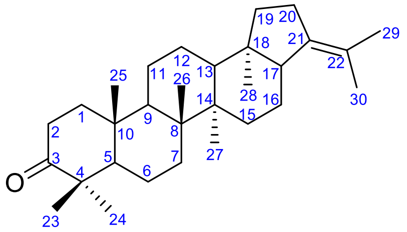

11

To the solution of hydroxyhopanone (**1**) (2.0 g, 4.7 mmol, 1.0 eq.) in anhydrous toluene (200 mL) and CH_2Cl_2 (10 mL) with 3 Å MS was added $PdCl_2$ (400 mg, 2.3 mmol. 0.5 eq.). The reaction was kept under an atmosphere of argon at 70 °C for 5 hours. The suspension was filtered through Celite and the filtrate was concentrated. The residual material was subjected to Ag-coated silica chromatography (hexanes: Et_2O = 20:1) to afford the desired product 3-oxo-diploptene (**7**) (0.8 g, 40%) as a colorless solid and the isomer **11** (0.3 g, 30%) as a colorless solid, which were further purified by crystallization, separately, from CH_2Cl_2 and hexane.

Compound **7**: R_f 0.33 (normal TLC, hexanes: diethyl ether = 9: 1) and 0.14 (Silver-coated TLC, hexanes: diethyl ether = 9: 1); $[\alpha]_D^{20}$ = +84 (*c* = 0.25, CH_2Cl_2); m.p. 210-212 °C (CH_2Cl_2-hexane); (Lit. 194 °C (acetone) (Dunstan et al., 1957)); ν_{max} (ATR)/cm^{-1} 2946, 2862 ($\nu_{C\text{-}H}$), 1705 ($\nu_{C=O}$), 1645, 1632 ($\nu_{C=C}$), 1444, 1376 ($\nu_{C\text{-}H}$), 1005 ($\nu_{C\text{-}O}$), 887 ($\nu_{C\text{-}H}$); ^{1}H NMR (500 MHz, CD_2Cl_2) δ 0.73 (s, 3H, H-28), 0.93 (s, 3H, H-25), 0.95 (s, 3H, H-27), 1.00 (s, 3H, H-23), 1.01 (s, 3H, H-26), 1.04 (s, 3H, H-24), 1.01-1.09 (m, 1H, H-19*a*), 1.22-1.69 (m, 18 H, H-15*a*, H-7*e*, H-5, H-9, H-1*e*, H-13, H-17, H-15*e*, H-12*a*, H-11*e*, H-6*a*, H-12*e*, H-11*a*, H-6*e*, H-7*a*, H-16*a*, H-19*e*, H-16*e*), 1.75 (s, 3H, H-30), 1.76-1.89 (m, 2H, H-20), 1.88-1.96 (m, 1H, H-1*a*), 2.31-2.39 (m, 1H, H-2*e*), 2.43-2.52 (m, 1H, H-2*a*), 2.65-2.74 (m, 1H, H-21), 4.77 (s, 2H, H-29); ^{13}C NMR (125 MHz, CD_2Cl_2); δ 15.9 (C-25), 16.3 (C-28), 16.5 (C-26), 16.7 (C-27), 20.1 (C-6), 21.3 (C-23), 21.9 (C-12), 22.0 (C-16), 24.3(C-11), 25.1 (C-30), 26.6 (C-24), 27.7 (C-20), 33.0 (C-7), 34.0 (C-15), 34.5 (C-2), 37.2 (C-10), 39.9 (C-1), 42.0 (C-8), 42.5 (C-14), 42.2 (C-19), 45.1 (C-18), 46.9 (C-21), 47.5 (C-4), 49.9 (C-13), 50.0 (C-9), 55.1 (C-5), 55.2 (C-17), 110.2 (C-29), 149.1 (C-22), 217.7 (C-3); HRMS *m/z* (FI) [Calculated for $C_{30}H_{48}O$ 424.3705; found 424.3698].

Compound **11**: R_f 0.32 (normal TLC, hexanes: diethyl ether = 9: 1) and 0.24 (Silver-coated TLC, hexanes: diethyl ether = 9: 1); $[\alpha]_D^{20}$ = +54 (*c* = 0.5, CH_2Cl_2) (literature: +67 (Dunstan et al., 1957)); m.p. 205-208 °C (CH_2Cl_2-hexane) (Lit. 189-193 °C, acetone) (Dunstan et al., 1957)); ν_{max} (ATR)/cm^{-1} 2972, 2945, 2925, 2865 ($\nu_{C\text{-}H}$), 1703 ($\nu_{C=O}$), 1680 ($\nu_{C=C}$), 1459, 1383, 1372 ($\nu_{C\text{-}H}$), 1001 ($\nu_{C\text{-}O}$); ^{1}H NMR (500 MHz, CD_2Cl_2) δ 0.59 (s, 3H, H-28), 0.93 (s, 3H, H-25), 0.98 (s, 3H, H-27), 1.00 (s, 3H, H-23), 1.02 (s, 3H, H-26),

1.04 (s, 3H, H-24), 1.27 (dt, 1H, *J* 3.3, 13.2 Hz, H-15*a*), 1.29-1.56 (m, 14H, H-7*e*, H-5, H-9, H-12*a*, H-1*a*, H-15*e*, H-6*a*, H-11*e*, H-11*a*, H-13, H-6*e*, H-7*a*, H-19*e*, H-12*e*), 1.57 (s, 3H, H-29/30), 1.61 (td, 1H, *J* 3.7, 12.6 Hz, H-16*a*), 1.73 (s, 3H, H-30/29), 1.74-1.80 (m, 1H, H-17), 1.89-1.96 (m, 1H, H-1*e*), 2,07 (dd, 1H, *J* 9.9, 16.3 Hz, H-20*a*), 2.13-2.26 (m, 2H, H-16*e*, H-20*e*) 2.30-2.39 (m, 1H, H-2*e*), 2,43-2.53 (m, 1H, H-2a); ^{13}C NMR (125 MHz, CD_2Cl_2) δ 14.8 (C-28), 15.9 (C-25), 16.5 (C-26), 16.6 (C-27), 19.5 (C-29/30), 20.1 (C-6), 21.3 (C-23), 21.9 (C-12), 22.9 (C-30/29), 23.7 (C-16), 24.0 (C-11), 26.7 (C-24), 28.7 (C-20), 33.0 (C-7), 33.2 (C-15), 34.6 (C-2), 37.2 (C-10), 39.4 (C-19), 39.9 (C-1), 41.9 (C-8), 42.0 (C-14), 44.7 (C-18), 47.6 (C-4), 48.6 (C-13), 50.1 (C-9), 55.2 (C-5), 56.3 (C-17), 120. 9 (C-22), 135.9 (C-21), 217.7 (C-3); HRMS *m/z* (FI) [Calculated for $C_{30}H_{48}O$ 424.3705; found 424.3720].

Diploptene (8)

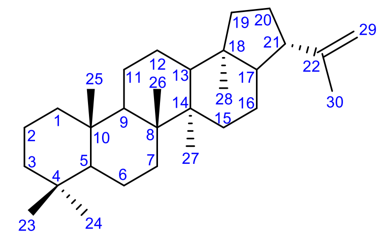

Compound **7** (50 mg, 0.12 mmol, 1.0 eq.) was dissolved in toluene (20 mL) and 4-toluenesulfonyl hydrazide (42 mg, 0.22 mmol, 1.8 eq.) was added. The solution was heated under reflux with a Soxhlet condenser filled with activated 3 Å molecular sieves for 2 hours. After this time, the solution was cooled to RT and $NaBH_4$ (20 mg, 0.53 mmol, 4.4 eq.) and methanol (5 mL) were added in two portions over a period of 10 min. After heating under reflux for a further 30 min, the solution was cooled down to RT and concentrated on a rotary evaporator. The residual material was subjected to silica chromatography (hexane) to afford diploptene (**8**) as a colorless solid (41 mg, 82%) that was crystallized from hexane and methanol to give small needles: R_f 0.91 (normal TLC, hexanes: diethyl ether = 9: 1) and 0.59 (Silver-coated TLC, hexanes: diethyl ether = 9: 1); $[\alpha]_D^{20}$ = +60 (*c* = 0.25, CH_2Cl_2) (Lit. +56.7 (*c* = 0.10, $CHCl_3$ (Corbett and Smith, 1967)); m.p. 199-201 °C (CH_2Cl_2-hexane) (Lit. 205 °C (hexane-ethanol) (Corbett and Smith, 1967) & 212-215 °C (Tsuda et al., 1967)); ν_{max} (ATR)/cm^{-1} 2943, 2925, 2867 (ν_{C-H}), 1645 ($\nu_{C=C}$), 1458, 1388 (ν_{C-H}), 886, 801 (ν_{C-H}); ^{1}H NMR (500 MHz, CD_2Cl_2) δ 0.72 (s, 3H, H-28), 0.70-0.77 (m, 2H, H-5, H-1*a*), 0.80 (s, 3H, H-23), 0.82 (s, 3H, H-25), 0.84 (s, 3H, H-24), 0.95 (s, 3H, H-27), 0.97 (s, 3H, H-26), 1.00-1.09 (m, 1H, H-19*a*), 1.13 (td, 1H, *J* 4.8, 14.0 Hz, H-3*a*), 1.17-1.30 (m, 3H, H-7*e*, H-15*a*, H-9), 1.30-1.43 (m, 8H, H-12*a*, H-6*a*, H-3*e*, H-2*e*, H-13, H-15*e*, H-11*e*, H-17), 1.43-1.68 (m, 9 H, H-11*a*, H-16*a*, H-7*a*, H-6*e*, H-12*e*, H-2*a*, H-19*e*, H-16*e*, H-1*e*), 1.74 (s, 3H, H-30), 1.76-1.90 (m, 2H, H-20), 2.64-

2.74 (m, 1H, H-21), 4.77 (s, 2H, H-29); ^{13}C NMR (100 MHz, CD_2Cl_2) δ 16.0 (C-25), 16.3 (C-28), 16.8 (C-26), 16.9 (C-27), 19.1 (C-2, C-6), 21.3 (C-12), 21.7 (C-23), 22.1 (C-16), 24.4 (C-11), 25.1 (C-30), 27.8 (C-20), 33.55 (C-4), 33.56 (C-7), 33.64 (C-24), 34.0 (C-15), 37.8 (C-10), 40.7 (C-1), 42.2 (C-19), 42.3, 42.4 (C-8, C-14), 42.5 (C-3), 45.2 (C-18), 47.0 (C-21), 49.8 (C-13), 50.8 (C-9), 55.3 (C-17), 56.4 (C-5), 110.2 (C-29), 149.3 (C-22); HRMS *m/z* (FI) [Calculated for $C_{30}H_{50}$ 410.3913; found 410.3913].

2,2-D$_2$-3-Oxo-diploptene (9)

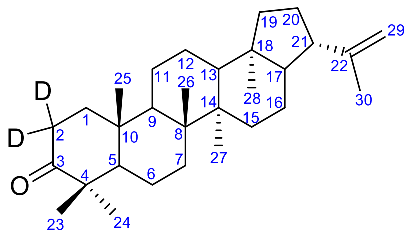

Compound **7** (100 mg, 0.24 mmol, 1.0 eq.) was dissolved in CD_2Cl_2 (5.0 mL), MeOD (1.67 mL) and D_2O (0.25 mL) in a glass flask and NaOD in D_2O (40 wt.%, 13 µL, 0.77 eq.) was added. The reaction was kept overnight under stirring and then quenched by a dry carbon dioxide flow. The deuterated product was extracted by an ether/water extraction. The organic fraction was dried over magnesium sulfate and filtered. The solvent was removed on a rotary evaporator to afford product **9** as colorless crystals in 100% yield (100 mg): R_f 0.33 (normal TLC, hexanes: diethyl ether = 9: 1) and 0.14 (Silver-coated TLC, hexanes: diethyl ether = 9: 1); $[\alpha]_D^{20}$ = +78 (*c* = 0.25, CH_2Cl_2); m.p. 219-221 °C (CH_2Cl_2); ν_{max} (ATR)/cm^{-1} 2947, 2927, 2856 ($\nu_{C\text{-}H}$), 1703 ($\nu_{C=O}$), 1647, 1632 ($\nu_{C=C}$), 1444, 1262 ($\nu_{C\text{-}H}$), 1082 ($\nu_{C\text{-}O}$), 801 ($\nu_{C\text{-}H}$); ^1H NMR (500 MHz, CD_2Cl_2) δ 0.73 (s, 3H, H-28), 0.93 (s, 3H, H-25), 0.95 (s, 3H, H-27), 1.00 (s, 3H, H-23), 1.01 (s, 3H, H-26), 1.04 (s, 3H, H-24), 1.02-1.09 (m, 1H, H-19*a*), 1.22-1.69 (m, 18 H, H-15*a*, H-7*e*, H-5, H-9, H-1*a*, H-13, H-17, H-15*e*, H-12*a*, H-11*e*, H-6*a*, H-12*e*, H-11*a*, H-6*e*, H-7*a*, H-16*a*, H-19*e*, H-16*e*), 1.75 (s, 3H, H-30), 1.76-1.89 (m, 2H, H-20), 1.88-1.95 (m, 1H, H-1*e*), 2.66-2.74 (m, 1H, H-21), 4.78 (s, 2H, H-29); ^{13}C NMR (125 MHz, CD_2Cl_2) δ 15.9 (C-25), 16.3 (C-28), 16.6 (C-26), 16.8 (C-27), 20.1 (C-6), 21.3 (C-24), 21.9 (C-12), 22.0 (C-16), 24.3 (C-11), 25.1 (C-30), 26.6 (C-23), 27.7 (C-20), 33.0 (C-7), 34.1 (C-15), 37.2 (C-10), 39.8 (C-1), 42.0, 42.5 (C-8, C-14), 42.3 (C-19), 45.1 (C-18), 46.9 (C-21), 47.6 (C-4), 50.0 (C-13), 50.1 (C-9), 55.2 (C-5), 55.3 (C-17), 110.2 (C-29), 149.2 (C-22), 217.9 (C-3); HRMS *m/z* (FI) [Calculated for $C_{30}H_{46}D_2O$ 426.3831; found 426.3822].

2,2,3,3-D_4-diploptene (10)

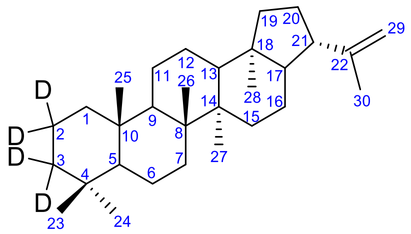

Compound **9** (50 mg, 0.12 mmol, 1.0 eq.) was dissolved in toluene (20 mL). 4-Toluenesulfonyl hydrazide (44 mg, 0.23 mmol, 1.9 eq.) was added to the solution, followed by heating under reflux with a Soxhlet condenser filled with activated 3 Å molecular sieves. After 2 hours, the solution was cooled to RT. $NaBD_4$ (20 mg, 0.48 mmol, 4.0 eq.) and MeOD (5 mL) were added in two portions over a period of 10 min. After heating under reflux for a further 30 min, the solution was cooled to RT and concentrated on a rotary evaporator. The residual material was subjected to silica chromatography (hexane) to afford compound **10** as a cololess solid (45 mg, 90%) that was crystallized from hexane and mechanol to give small needles: R_f 0.91 (normal TLC, hexanes: diethyl ether = 9: 1) and 0.59 (silver-coated TLC, hexanes: diethyl ether = 9: 1); $[\alpha]_D^{20}$ =+60 (*c* = 0.25, CH_2Cl_2); m.p. 209-210 °C (hexane-methanol); ν_{max} (ATR)/cm^{-1} 2944, 2925, 2894, 2864 (ν_{C-H}), 1645, 1633 ($\nu_{C=C}$), 1444, 1377 (ν_{C-H}), 886, 750 (ν_{C-H}); ^1H NMR (500 MHz, CD_2Cl_2): δ 0.72 (s, 3H, H-28), 0.74-0.79 (m, 2H, H-5, H-1*a*), 0.79 (s, 3H, H-23), 0.82 (s, 3H, H-25), 0.84 (s, 3H, H-24), 0.95 (s, 3H, H-27), 0.97 (s, 3H, H-26), 0.99-1.11 (m, 1H, H-19*a*), 1.17-1.44 (m, 9H, H-7*e*, H-15*a*, H-9, H-12*a*, H-6*a*, H-13, H-15*e*, H-17, H-11*e*), 1.43-1.68 (m, 8 H, H-16*a*, H-11*a*, H-7*a*, H-6*e*, H-12*e*, H-19*e*, H-16*e*, H-1*e*), 1.75 (s, 3H, H-30), 1.76-1.90 (m, 2H, H-20), 2.64-2.73 (m, 1H, H-21), 4.77 (s, 2H, H-29); ^{13}C NMR (126 MHz, CD_2Cl_2): 16.0 (C-25), 16.3 (C-28), 16.8 (C-26), 16.9 (C-27), 19.1 (C-2, C-6), 21.3 (C-12), 21.7 (C-23), 22.1 (C-16), 24.4 (C-11), 25.1 (C-30), 27.8 (C-20), 33.3 (C-4), 33.5 (C-24), 33.6 (C-7), 34.0 (C-15), 37.7 (C-10), 40.4 (C-1), 40.5 (C-1*), 42.2 (C-19), 42.3 (C-8), 42.4 (C-14), 45.2 (C-18), 46.9 (C-21), 49.8 (C-13), 50.8 (C-9), 55.3 (C-17), 56.4 (C-5), 110.2 (C-29), 149.3 (C-22); HRMS *m/z* (FI) [Calculated for $C_{30}H_{46}D_4$ 414.4164; found 414.4161].

C1 of the tri-deuterated compound (C1:C1= ~3:1). Calculated with the signals in mass spec with Force Ionization (FI) D4:D3:D2 = 65:31:4.

3-Oxo-17-ene-deoxy-hopane (12)

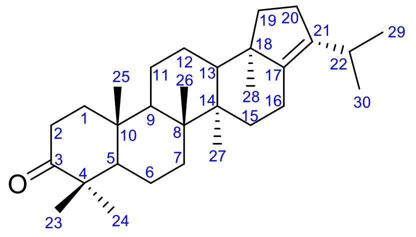

Refer to **Screening of conditions for better production of 7** (condition 11) for experimental procedures. Characterization of compound **12**: R_f 0.34 (normal TLC, hexanes: diethyl ether = 9: 1) and 0.22 (silver-coated TLC, hexanes: diethyl ether = 9: 1); $[\alpha]_D^{20}$ = +63 (*c* = 0.1, CH_2Cl_2); m.p. 195-198 °C (CH_2Cl_2); ν_{max} (ATR)/cm^{-1} 2989, 2944, 2855 (ν_{C-H}), 1706 ($\nu_{C=O}$), 1663, 1632 ($\nu_{C=C}$), 1459, 1448, 1379 (ν_{C-H}), 1002 (ν_{C-O}); ^{1}H NMR (500 MHz, CD_2Cl_2) δ 0.59 (s, 3H, H-28), 0.92 (d, 3H, *J* 7.8 Hz, H-30), 0.93 (s, 3H, H-25), 0.97 (s, 3H, H-26), 0.98 (d, 3H, *J* 7.1 Hz, H-29), 1.00 (s, 3H, H-23), 1.05 (s, 3H, H-24), 1.06 (s, 3H, H-27), 1.27-1.60 (m, 15 H, H-15*a*, H-15*e*, H-19*a*, H-5, H-7*e*, H-11*e*, H-12*a*, H-9, H-1*a*, H-12*e*, H-6*a*, H-13, H-6*e*, H-7*a*, H-11*a*), 1.67 (dd, 1H, *J* 7.5, 11.7 Hz, H-19*e*), 1.88-1.99 (m, 2H, H-16*a*, H-1*e*), 2.12 (dd, 1H, *J* 9.4, 15.3 Hz, H-20*a*), 2.16-2.26 (m, 1H, H-20*e*), 2.29 (dt, 1H, *J* 3.6, 14.3 Hz, H-16*e*), 2.33-2.41 (m, 1H, H-2*e*), 2.42-2.52 (m, 1H, H-2*a*), 2.60-2.71 (m, 1H, H-22); ^{13}C NMR (126 MHz, CD_2Cl_2) δ 15.0 (C-27), 16.2 (C-26), 16.3 (C-25), 19.2 (C-28), 20.1 (C-6), 20.2 (C-16), 21.2 (C-23), 21.4 (30), 22.0 (C-29), 22.3 (C-11), 24.4 (C-12), 26.8 (C-22, C-24), 27.8 (C-20), 32.2 (C-15), 33.2 (C-7), 34.5 (C-2), 37.3 (C-10), 40.1 (C-1), 42.0 (C-8), 42.4 (C-14), 42.1 (C-19), 47.6 (C-18), 49.9 (C-13), 50.2 (C-4), 50.5 (C-9), 55.2 (C-5), 136.6 (C-21), 140.3 (C-17), 217.9 (C-3); HRMS *m/z* (FI) [Calculated for $C_{30}H_{48}O$ 424.3705; found 424.3539].

Bacteriohopanetetrol (BHT, 13)

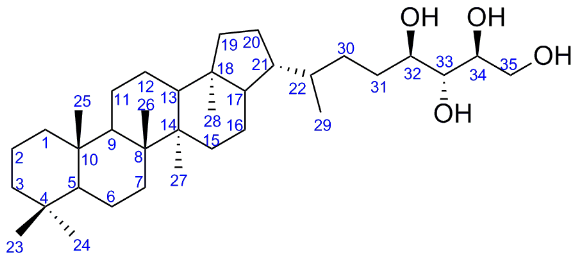

Refer to Refer to **Large scale total lipid extraction and Hopanoid purification by silica gel and HPLC** (Page S9) for purification procedure. The optimized procedure to dissolve **13** NMR experiments was as follows. MeOD (1 mL) was added to BHT (7.2 mg), followed by vortex and solication. A slightly white gel was formed after overnight stand on the bench at RT. $CDCl_3$ (1 mL each time) was added slowly to the gel, followed by strong vortex, sonication and short-time heating at 40 ºC repeatedly. After the addition of repetition of this step 5 times, a clear and colorless solution was formed. Compound **13**: R_f 0.88 (water: 2-propanol: EtOAc = 1:8:10); $[\alpha]_D^{20}$ = +58.3 (*c*

0.12, $CDCl_3$: MeOD = 5: 1); ν_{max} (ATR)/cm^{-1} 3340 (ν_{N-H}, ν_{O-H}), 2944, 2864 (ν_{C-H}), 1442, 1378 (ν_{C-H}), 1085, 1014 (ν_{C-O}); ^{1}H NMR (500 MHz, MeOD:$CDCl_3$ = 1:5) δ 0.52-0.61 (m, 1H, H-5), 0.59 (s, 3H, H-28), 0.65 (dd, 1H, *J* 3.4, 13.1 Hz, H-1*a*), 0.68 (s, 3H, H-23), 0.71 (s, 3H, H-25), 0.74 (s, 3H, H-24), 0.74-0.81 (m, 1H, H-19*a*), 0.81-0.86 (m, 9H, H-26, H-27, H-29), 0.94-1.06 (m, 1H, H-3*a*), 1.07-1.32 (m, 13H, H-7*e*, H-15*a*, H-30a, H-9, H-17, H-31a, H-12*a*, H-13, H-3*e*, H-15*e*, H-6*a*, H-2*e*, H-11*e*), 1.32-1.51 (m, 11H, H-11*a*, H-7*e*, H-30b, H-31b, H-2*a*, H-6*e*, H-12*e*, H-22, H-19*e*, H-16*a*, H-20*a*), 1.50-1.57 (m, 1H, H-1*e*), 1.58-1.75 (m, 3H, H-16*e*, H-21, H-20*e*), 3.37 (t, 1H, *J* 6.3 Hz, H-33), 3.49-3.68 (m, 4H, H-32, H-34, H-35); ^{13}C NMR (125 MHz, MeOD:$CDCl_3$ = 1:5) δ 16.08 (C-28), 16.14 (C-25), 16.7 (C-27), 16.8 (C-26), 18.97 (C-6), 18.99 (C-2), 20.2 (C-29), 21.3 (C-12), 21.8 (C-23), 23.1 (C-16), 24.3 (C-11), 28.0 (C-20), 29.6 (C-31), 31.9 (C-30), 33.5 (C-4), 33.59 (C-24), 3.63 (C-7), 34.0 (C-15), 37.2 (C-22), 37.7 (C-10), 40.6 (C-1), 41.9 (C-19), 42.0 (C-8), 42.1 (C-14), 42.4 (C-3), 44.7 (C-18), 46.6 (C-21), 49.6 (C-13), 50.8 (C-9), 54.8 (C-17), 56.5 (C-5), 63.6 (C-35), 73.3 (C-34), 74.1 (C-32), 74.8 (C-33); HRMS *m/z* (ES+) [Calculated for $C_{35}H_{62}NaO_4^+$ 569.4540; found 569.4547].

32,33,34,35-*tetra*-*O*-Acetyl bacteriohopanetetrol (14)

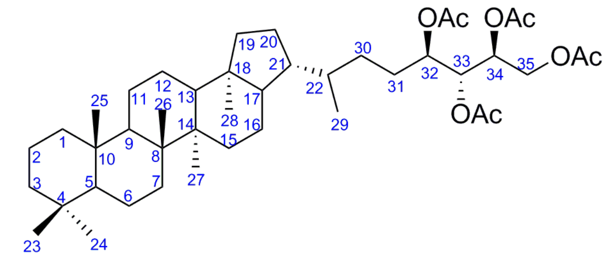

BHT (**13**) (2.0 mg, 3.7 μmol) was dissolved in pyridine (0.5 mL). Acetic anhydride (3 drops, ~12 μL) was added. The reaction was kept under stirring for 6 hours. The solvent was removed on a rotary evaporator. The residual material was subjected to silica chromatography (petroleum: diethyl ether = 1: 4) to afford compound **14** as a wax-like material (2.1 mg, 81%): R_f 0.40 (hexanes: diethyl ether = 1: 1); $[\alpha]_D^{20}$ = +52 (*c* = 0.075, diethyl ether); ν_{max} (ATR)/cm^{-1} 2942, 2867 (ν_{C-H}), 1747 ($\nu_{C=O}$), 1220 (ν_{C-O}); ^{1}H NMR (700 MHz, $CDCl_3$) δ 0.69 (s, 3H, H-28), 0.71 (dd, 1H, *J* 1.5, 12.3 Hz, H-5), 0.76 (dd, *J* 3.5, 12.9 Hz, H-1*a*), 0.79 (s, 3H, H-23), 0.81 (s, 3H, H-25), 0.85 (s, 3H, H-24), 0.87-0.91 (m, 1H, H-19*a*), 0.91 (d, *J* 6.4 Hz, H-29), 0.94 (s, 3H, H-27), 0.95 (s, 3H, H-26), 1.11 (m, 2H, H-30a), 1.13 (td, *J* 4.0, 13.3 Hz, H-3*a*), 1.19-1.67 (m, 24H, H-7*e*, H-15*a*, H-9, H-17, H-12*a*, H-6*a*, H-13, H-15*e*, H-3*e*, H-6*e*, H-11*e*, H-30b, H-20*a*, H-11*a*, H-22, H-7*a*, H-2*e*, H-31a, H-19*e*, H-16*a*, H-12*e*, H-2*a*, H-31b, H-1*e*), 1.68-1.80 (m, 3H, H-16*e*, H-20*e*, H-21), 2.05 (s, 3H, C-35-OCOC*H*$_3$), 2.07 (s, 3H, C-32-OCOC<u>*H*</u>$_3$), 2.079 (s, 3H, C-33-OCOC<u>*H*</u>$_3$), 2.084 (s, 3H, C-34-OCOC<u>*H*</u>$_3$), 4.15 (dd, 1H, *J* 6.7, 12.2 Hz, H-35a), 4.38 (dd, 1H, *J* 2.7, 12.2 Hz, H-35b), 5.03 (dt, 1H, *J* 3.8, 9.3 Hz, H-32), 5.23 (dd, 1H, *J* 4.4, 5.4 Hz, H-33), 5.25-5.29 (m, 1H, H-34); ^{13}C NMR (176 MHz, $CDCl_3$) δ 16.0 (C-28), 16.1 (C-25), 16.7 (C-27), 16.8 (C-26), 18.9 (C-2, C-6), 20.2 (C-29), 20.95 (C-33-OCO<u>*C*</u>H$_3$), 21.00 (C-34-OCO<u>*C*</u>H$_3$), 21.12 (C-32-OCO<u>*C*</u>H$_3$), 21.20 (C-35-OCO<u>*C*</u>H$_3$), 21.17 (C-12), 21.8 (C-24), 23.0 (C-16), 24.2 (C-11), 26.4 (C-31), 27.6 (C-20), 31.1 (C-30), 33.47 (C-

4), 33.5 (C-7), 33.6 (C-23), 34.0 (C-15), 36.3 (C-22), 37.6 (C-10), 40.5 (C-1), 41.8 (C-19), 41.9 (C-8), 42.0 (C-14), 42.3 (C-3), 44.6 (C-18), 46.1 (C-21), 49.5 (C-13), 50.6 (C-9), 54.6 (C-17), 56.4 (C-5), 62.4 (C-35), 69.8 (C-34), 71.9 (C-32), 72.2 (C-33), 169.9 (C-33-O*C*OCH$_3$), 170.2 (C-34-O*C*OCH$_3$), 170.6 (C-32-O*C*OCH$_3$), 170.9 (C-35-O*C*OCH$_3$); HRMS *m/z* (ES+): [Calculated for $C_{43}H_{70}NaO_8^+$ 737.4963; found 737.4980]; LRMS *m/z* (ES+) 737.5 ([M+Na]$^+$, 100%).

2-Methyl bacteriohopanetetrol (2Me-BHT, 15)

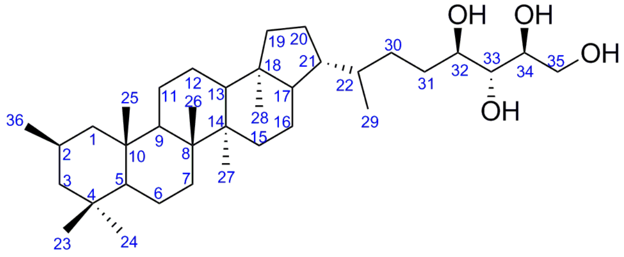

Refer to **Large scale total lipid extraction and Hopanoid purification by silica gel and HPLC** (Page S9) for purification procedure. The optimum solvent to dissolve compound **15** for NMR experiments was found to be THF. The purity of the sample was ~90%, judged by signals in the ^{1}H NMR spectrum. Trials on further cleaning up the samples were not successful. Compound **15**: R_f 0.88 (water: 2-propanol: EtOAc = 1:8:10); $[\alpha]_D^{20}$ = +33 (*c* 0.1, THF); ν_{max} (ATR)/cm^{-1} 3346 (ν_{N-H}, ν_{O-H}), 2927 (ν_{C-H}), 1457 (ν_{C-H}), 1066 (ν_{C-O}); ^{1}H NMR (500 MHz, D$_8$-THF) δ 0.76 (s, 3H, H-28), 0.83 (d, 3H, *J* 6.4 Hz, H-36), 0.85 (s, 3H, H-25), 0.86-0.92 (m, 1H, H-1*a*), 0.90 (s, 3H, H-24), 0.89-0.95 (m, 1H, H-19*a*), 0.93 (s, 3H, H-23), 0.95 (d, 3H, H-29), 0.977 (s, 3H, H-27), 0.980(s, 3H, H-26), 1.14 (bd, 1H, *J* 12.6 Hz, H-3*a*), 1.16-1.35 (m, 6H, H-30a, H-7*e*, H-3*e*, H-15*a*, H-17, H-9), 1.36-1.66 (m, 17H, H-13, H-15*e*, H-12*a*, H-11*e*, H-6*a*, H-6*e*, H-31a, H-7*a*, H-11*a*, H-1*e*, H-12*e*, H-22, H-30b, H-19*e*, H-31b, H-16*a*, H-20*a*), 1.64-1.71 (m, 1H, H-2), 1.71-1.87 (m, 3H, H-16*e*, H-21, H-20*e*), 3.30 (t, 1H, *J* 8.5 Hz, H-33), 3.48-3.68 (m, 3H, H-35a, H-32, H-34), 3.65 (dd, 1H, *J* 3.2, 9.8 Hz, H-35b); ^{13}C NMR (125 MHz, MeOD:CDCl$_3$ = 1:5) δ 15.8 (C-28), 16.0 (C-27), 16.3 (C-26), 19.9 (C-6, C-29), 21.7 (C-23), 21.9 (C-12), 22.8 (C-36), 23.1 (C-16), 24.2 (C-11), 24.7 (C-2), 25.9 (C-25), 27.6 (C-20), 29.3 (C-31), 30.9 (C-24), 31.5 (C-30), 32.4 (C-4), 32.5 (C-7), 33.7 (C-15), 36.9 (C-22), 37.8 (C-10), 41.65 (C-8), 41.70 (C-19), 41.85 (C-14), 44.3 (C-18), 45.1 (C-3), 46.2 (C-21), 49.6 (C-5), 49.7 (C-1, C-13), 50.9 (C-9), 54.5 (C-17), 63.2 (C-35), 72.9 (C-34), 73.7 (C-32), 74.4 (C-33). HRMS *m/z* (ES+) [Calculated for $C_{36}H_{64}NaO_4^+$ 583.4697; found 583.4705].

References

Bligh, E. G., and Dyer, W. J., 1959, A Rapid Method of Total Lipid Extraction and Purification: Canadian Journal of Biochemistry and Physiology, v. 37, no. 8, p. 911-917.

Bose, A., and Newman, D. K., 2011, Regulation of the phototrophic iron oxidation (pio) genes in Rhodopseudomonas palustris TIE-1 is mediated by the global regulator, FixK: Molecular Microbiology, v. 79, no. 1, p. 63-75.

Corbett, R. E., and Smith, R. A. J., 1967, Lichens and Fungi .4. Rearrangements at C-21 in Hopane Series: Journal of the Chemical Society C-Organic, no. 17, p. 1622-&.

Dunstan, W. J., Fazakerley, H., Halsall, T. G., and Jones, E. R. H., 1957, The chemistry of the triterpenes and related compounds. part XXXII. the chemistry of hydroxyhopanone: Croatica Chemica Acta, v. 29.

Isaac, G., McDonald, S., and Astarita, G., 2011, Lipid Separation using UPLC with Charged Surface Hybrid Technology: Waters Corporation Application Note, v. 720004107en.

Khan, S. R., Gaines, J., Roop, R. M., and Farrand, S. K., 2008, Broad-host-range expression vectors with tightly regulated promoters and their use to examine the influence of TraR and TraM expression on Ti plasmid quorum sensing: Applied and Environmental Microbiology, v. 74, no. 16, p. 5053-5062.

Korstanje, T. J., de Waard, E. F., Jastrzebski, J. T. B. H., and Gebbink, R. J. M. K., 2012, Rhenium-Catalyzed Dehydration of Nonbenzylic and Terpene Alcohols to Olefins: Acs Catalysis, v. 2, no. 10, p. 2173-2181.

Pangborn, A. B., Giardello, M. A., Grubbs, R. H., Rosen, R. K., and Timmers, F. J., 1996, Safe and convenient procedure for solvent purification: Organometallics, v. 15, no. 5, p. 1518-1520.

Quandt, J., and Hynes, M. F., 1993, Versatile Suicide Vectors Which Allow Direct Selection for Gene Replacement in Gram-Negative Bacteria: Gene, v. 127, no. 1, p. 15-21.

Tsuda, Y., Isobe, K., Fukushim.S, Ageta, H., and Iwata, K., 1967, Final Clarification of Saturated Hydrocarbons Derived from Hydroxyhopanone Diploptene Zeorin and Dustanin: Tetrahedron Letters, no. 1, p. 23-&.

Welander, P. V., Doughty, D. M., Wu, C. H., Mehay, S., Summons, R. E., and Newman, D. K., 2012, Identification and characterization of Rhodopseudomonas palustris TIE-1 hopanoid biosynthesis mutants: Geobiology, v. 10, no. 2, p. 163-177.
